# *DREB* Genes from Common Bean (*Phaseolus vulgaris* L.) Show Broad to Specific Abiotic Stress Responses and Distinct Levels of Nucleotide Diversity

**DOI:** 10.1155/2019/9520642

**Published:** 2019-05-02

**Authors:** Enéas Ricardo Konzen, Gustavo Henrique Recchia, Fernanda Cassieri, Danielle Gregorio Gomes Caldas, Jorge C. Berny Mier y Teran, Paul Gepts, Siu Mui Tsai

**Affiliations:** ^1^Cell and Molecular Biology Laboratory, Center for Nuclear Energy in Agriculture, University of Sao Paulo, Av. Centenário 303, Piracicaba, SP CEP 13.400-970, Brazil; ^2^Department of Plant Sciences/MS1, University of California, 1 Shields Av., Davis, CA 95616-8780, USA

## Abstract

We analyzed the nucleotide variability and the expression profile of *DREB* genes from common bean, a crop of high economic and nutritional value throughout the world but constantly affected by abiotic stresses in cultivation areas. As *DREB* genes have been constantly associated with abiotic stress tolerance, we systematically categorized 54 putative *PvDREB* genes distributed in the common bean genome. It involved from AP2 domain location and amino acid conservation analysis (valine at the 14^th^ position) to the identification of conserved motifs within peptide sequences representing six subgroups (A-1 to A-6) of PvDREB proteins. Four genes (*PvDREB1F*, *PvDREB2A*, *PvDREB5A*, and *PvDREB6B*) were cloned and analyzed for their expression profiles under abiotic stresses and their nucleotide and amino acid diversity in genotypes of Andean and Mesoamerican origin, showing distinct patterns of expression and nucleotide variability. *PvDREB1F* and *PvDREB5A* showed high relative inducibilities when genotypes of common bean were submitted to stresses by drought, salt, cold, and ABA. *PvDREB2A* inducibility was predominantly localized to the stem under drought. *PvDREB6B* was previously described as an A-2 (*DREB2*) gene, but a detailed phylogenetic analysis and its expression profile clearly indicated it belongs to group A-6. *PvDREB6B* was found as a cold- and dehydration-responsive gene, mainly in leaves. Interestingly, *PvDREB6B* also showed a high nucleotide and amino acid diversity within its coding region, in comparison to the others, implicating in several nonsynonymous amino acid substitutions between Andean and Mesoamerican genotypes. The expression patterns and nucleotide diversity of each *DREB* found in this study revealed fundamental characteristics for further research aimed at understanding the molecular mechanisms associated with drought, salt, and cold tolerance in common bean, which could be performed based on association mapping and functional analyses.

## 1. Introduction

Abiotic stresses have a negative impact on plants, limiting their growth and survival. An immediate response triggered by plants under abiotic stresses is an increase in the synthesis of abscisic acid, leading to stomata closure and, thus, reducing the photosynthetic activity [1, 2]. As a defense mechanism, several genes are induced in order to adjust or circumvent the stresses. One category of genes primarily works for mechanic and osmotic adjustment, while another set is involved in a series of regulation processes for overcoming the stress conditions [3]. The latter group comprises several stress-inducible genes such as *NAC*, *bZIP*, *leucine-rich repeats* (*LRR*), and *EREBP/AP2* [4].

EREBP/AP2 proteins constitute a large superfamily, which has been divided into three families (AP2, RAV, and ERF) based on sequence similarity and the number of EREBP/AP2 domains [5]. The ERF family contains only one EREBP/AP2 domain and two subfamilies named CBF/DREB and ERF. The amino acids at positions 14^th^ and 19^th^ from the beginning of the EREBP/AP2 domain sequence have been considered for distinguishing DREB (in general valine and glutamic acid, respectively) from ERF (normally alanine and aspartic acid, respectively) proteins [6]. ERF proteins are primarily involved in responses to biotic stresses, such as pathogenesis, by recognizing the AGCCGCC *cis*-regulatory element, known as GCC box [7]. On the other hand, DREB proteins have a crucial role in the response of plants to abiotic stresses by recognizing the dehydration responsive element (DRE), which consists on the conserved motif A/GCCGAC [8]. DRE has been found essential for gene regulation due to dehydration [8], but since then, it has also been found in the promoter region of other drought-, salinity-, and cold-inducible genes [5, 6, 9].

*DREB* genes are usually divided into six subgroups (A-1-A-6). The general trend observed in *Arabidopsis* is that *DREB1/CBF* (A-1) genes are induced by low temperature, while *DREB2* genes (A-2) are involved in responses to osmotic stress (dehydration and salinity) [6, 10]. Overall, studies have shown that the expression of members of A-1 and A-2 subgroups is usually not mediated by ABA signaling in *Arabidopsis*. Conversely, *ABI4*, the only member of the A-3 subgroup, is involved in ABA and sugar signaling, lipid mobilization in embryos and germinating seeds, chloroplast functioning, and retrograde signaling [11]. The most studied members of the A-4 subgroup are *TINY*, which has been shown to be slightly cold-responsive [6], and *HARDY*, with low stress inducibility, but with potential for augmenting water use efficiency when overexpressed in rice [5, 12]. Moreover, studies suggested that A-4 genes are involved in the cross-talk between abiotic and biotic stress response by connecting DRE- and ERE- (ethylene-responsive element) mediated signaling pathways [13, 14]. Genes of the A-5 group from *Arabidopsis*, such as *RAP2.1*, exhibit induction by drought and cold stress [15]. In group A-6, *RAP2.4* (salt- and drought-responsive) [16] and *RAP2.4B* (heat-responsive) [17] are among the most studied.

Besides *Arabidopsis*, *DREB* genes have been isolated and characterized in several other plants. With detailed studies of *DREB* orthologs, the functional classification between A-1 and A-2 became less clear [3], as some A-2 genes have been discovered to be regulated by low temperature as well [18]. Moreover, some *DREB* genes either from A-1 and A-2 subgroups were found to be affected by ABA in other plants, such as soybean [19, 20].

With the release of several plant whole-genome sequences, genome-wide analyses have been performed to identify *ERF* and *DREB* genes of various species such as *A. thaliana* [5], *G. max* [21], *Malus domestica* [22], *Zea mays* [23], *Brassica rapa* [24], *Brassica oleracea* [25, 26], *Brassica napus* [27], *Vitis vinifera* [28], *Setaria italica* [29], *Eucalyptus grandis* [30], *Salix arbutifolia* [31], *Phyllostachys edulis* [32, 33], and *Syntrichia caninervis* [34].

In legumes, one work was devoted to a comparative analysis of the AP2/ERF and HSP90 gene families in chickpea, pigeon pea, *Medicago*, *Lotus*, and common bean [35]. As regards the ERF family of transcription factors, Kavas et al. [36] performed a general investigation of all representatives in common bean, also characterizing the *DREB* gene subfamily. Although general categorizations are already available for the common bean *ERF* subfamily, no further investigation has been done specifically for DREB transcription factors.

Common bean (*Phaseolus vulgaris*) is native to America, spreading from northern Mexico to northern Argentina [37, 38]. Its genetic diversity is structured in two major gene pools, Andean and Mesoamerican, from which the cultivated types were independently derived [39, 40]. To date, common bean is the most important grain legume for consumption, grown mainly in Latin America, Africa, and Asia. Bean grains are widely consumed in these areas since they are a source of proteins, vitamins, and minerals with a crucial role in nutrition [41, 42]. Several small farmers consider beans as a complimentary if not the basic food source, especially in Latin America and Africa. However, its production has been severely impaired by a lot of abiotic factors such as constant drought episodes [42], soil salinity [43], low or high temperatures [44], and nutritional deficiencies [45]. This problem becomes even more aggravated since most producers have little access or financial resources for irrigation and soil management [41]. Such scenario requires the development of efficient strategies toward breeding for abiotic stress tolerance improvement in common bean varieties throughout the world.

The release of the common bean genome [46] has opened multiple possibilities for studying the molecular mechanisms involved in the responses of abiotic stresses in the species. Moreover, the development of the 6K SNP BeadChip, BeanCAP Project [47], and other SNP libraries [48] has provided markers for studies with common bean populations with diverse goals [49, 50].

This work was driven to the identification of *DREB* genes in the common bean reference genome, isolating four genes and unraveling their responses under abiotic stresses. *In silico* analyses provided the categorization of 54 putative DREB members. Alignments, phylogenies, and motif predictions were generated to designate *DREB* genes, following several criteria based on *Arabidopsis* and other model plants. The SNP array developed for common bean was searched for the closest SNP to each one of the *PvDREB* genes, and diversity analysis was performed with a set of genotypes. Furthermore, we isolated four *PvDREB* (*PvDREB1F*, *PvDREB2A*, *PvDREB5A*, and *PvDREB6B*) genes and analyzed their nucleotide diversity and expression profiles under dehydration, salinity, low temperature, and abscisic acid treatment, providing insights for their application in breeding and engineering of stress tolerance in common bean.

## 2. Material and Methods

We performed a genome-wide categorization of the *DREB* gene family in common bean by following five basic criteria. First, we checked for the presence of one AP2 conserved domain along the protein structure (criterion 1). Second, ortholog relationships were verified among common bean, *A. thaliana*, and *G. max* AP2-containing sequences and *DREB* genes with defined nomenclature (criterion 2). Next, amino acid conservation was verified along the AP2 domain of predicted protein sequences (criterion 3). The fourth verification consisted on phylogenetic analyses and subgroup division establishments (criterion 4). Ultimately, conserved motifs were searched along the peptide sequences of all putative DREB proteins (criterion 5).

### 2.1. *In Silico* Search for AP2/ERF Proteins and Annotation

The first step was to identify all ERF proteins, which presumed the presence of at least one AP2 conserved domain. Phytozome (https://www.phytozome.net) [51] and GenBank (https://www.ncbi.nlm.nih.gov/genbank) databases were accessed to search all *P. vulgaris* peptide sequences containing the AP2 domain. The database showed 184 unigenes matching this search (Supplementary File 1). All peptides were double-checked on PFAM (https://pfam.sanger.ac.uk/) and SMART (http://smart.embl-heidelberg.de/) for the presence of AP2 or other domains. We only considered for further analysis those peptides presenting a single AP2 domain, which represents one basic aspect of the DREB family. Moreover, a local BLASTp was performed against *G. max* and *A. thaliana* with an *E*-value cutoff of 1 × 10^−5^ to identify domains and possible orthologs of *DREB* genes (Supplementary File 1).

### 2.2. Alignment and Phylogenetic Analyses

Alignments and phylogenetic analyses were performed with full-length peptide sequences from common bean. CLC Sequence Viewer version 6 (https://www.clcbio.com/) software was used for sequence alignment, considering default parameters. Global alignment was performed with ClustalW tool, and phylogenetic trees were generated using MEGA 6.0 [52] by the neighbor-joining algorithm with bootstrap analysis with 1000 permutations.

All AP2 domain-containing sequences from *P. vulgaris*, *A. thaliana*, and *G. max* were downloaded from Phytozome. In total, 57 ERF sequences were categorized as *DREB* genes in Arabidopsis genome [5]; those were retrieved from TAIR (https://www.arabidopsis.org/). Zhang et al. [21] categorized 98 ERF genes in soybean, of which 36 represented *DREB* genes. However, the analyses were performed before the genomic sequence of *G. max* had been released [53]. We used AP2-containing sequences from the current version of the soybean genome (available on Phytozome). Furthermore, the core sequences for DREB proteins already isolated and characterized for *A. thaliana* and soybean were accessed on GenBank. All these sequences were aligned and grouped with the neighbor-joining algorithm in order to verify which common bean sequences were more similar to the DREB proteins already known for the other species (Supplementary File 2).

### 2.3. Alignment of the AP2 Domain and Amino Acid Conservation

All putative DREB proteins were aligned to verify if they had conserved positions 14^th^ and 19^th^ from the beginning of the AP2 domain (positions were determined based on the original *Arabidopsis* sequences), respectively, with valine (V) and glutamic acid (E), which have been shown to be essential for DREB proteins' binding specificity, especially valine [6]. Sequences not following this criterion were discarded from the analysis. A new alignment and phylogenetic analysis were performed to confirm if the sequences matched orthologs from *G. max* and *Arabidopsis*.

### 2.4. Categorization of Putative DREB Members into Subgroups

The phylogenetic tree with all AP2-containing sequences from common bean, soybean, and Arabidopsis was analyzed to categorize all common bean DREB proteins into six groups (A-1 to A-6), based on previous references [5, 6, 21]. These groups were validated through an analysis of conserved motifs shared among sequences within the same group.

### 2.5. Protein Motif Search and In Silico Mapping

Conserved motif search was performed with the MEME tool [54]. Motif search criteria were based on previous studies [5, 21, 22], but we also determined the threshold for motif detection as the maximum number of motifs that could be detected without having a significant similarity among each other.

The genomic positions of the *PvDREB* genes were placed in a map that has been created using MapDraw [55]. Gene positions were checked on Phytozome, and those that were not separated by more than five gene loci over 100 kb were considered tandem duplicates [22].

### 2.6. Gene Ontology (GO) Annotation and Phyto Mine Expression Profiling

The GO annotation of the putative *DREB* genes was investigated through Blast2GO [56]. Expression profiles for each gene were obtained based on FPKM (fragments per kilobase of transcript per million mapped reads) values retrieved from Phyto Mine on Phytozome, searching the *P. vulgaris* genome. The Phyto Mine database shows a series of RNA-Seq data obtained for several plant organs in common bean (flower buds, pods, roots, leaves, stem, flowers, nodules, and young trifoliolates). All negative FPKM values were considered as zero expression values or nearly undetectable transcripts. A heat map was drawn in R, using the package gplots 2.17.0 and the function heat map.2 [57].

### 2.7. The Genes *PvDREB1F*, *PvDREB2A*, *PvDREB5A*, and *PvDREB6B*

We used *DREB* genes already characterized in *A. thaliana* to generate a gene expression profile compilation based on microarray data provided by Genevestigator analytical tool (https://genevestigator.com/gv/). Data were shown as heat maps in red/green coding, which were represented by log ratios (red representing upregulation and green downregulation—probe sets in a 22 k Affymetrix GeneChip) (Supplementary File 3). Ortholog genes in *P. vulgaris* were searched. In this step, genes representing the *DREB* subfamily were chosen for expression profiling.

Four genes were cloned and named *PvDREB1F* (KX151399 at the GenBank), *PvDREB2A* (KX151398), *PvDREB5A* (KX151397), and *PvDREB6B* (KX147642), based on phylogenetic analyses and comparisons with their orthologs in *A. thaliana*. Gene-specific primers (Supplementary File 4) were designed to clone the entire coding region of the four *DREB*. Genes were cloned from the common bean genotype BAT 477. DNA was extracted through a modified CTAB extraction protocol from Doyle [58]. PCR was prepared to 25 *μ*L containing 1x PCR reaction buffer (20 mM Tris-HCl pH 8.4, 50 mM KCl), 1.6 mM MgCl_2_, 0.12 mM dNTP, 0.2 *μ*M of each primer, and 1 U of Taq DNA polymerase (Invitrogen™). Amplification conditions were set as follows: initial denaturation at 94°C for 2 min; 35 cycles at 94°C for 30 s (denaturation), 59°C for 30 s (primer annealing), and 72°C (extension of fragments); and final extension at 72°C for 7 min. Fragments were gel-purified using GFX purification kit (GE Healthcare) and cloned into the p-GEM vector (Invitrogen) with thermo-competent JM109 *Escherichia coli* cells. Transformed colonies were analyzed by blue/white plaque assays, cultured in circle-growth medium, and purified. Sequencing was performed in ABI PRISM® 3130xl Sequencer equipment. Sequences were annotated using BLASTn, BLASTx, and BLASTp tools (NCBI/BLAST). Sequences were aligned to those available on Phytozome as a final check for their identity.

### 2.8. Plant Materials and Stress Treatments

Two sets of experiments were carried out for gene expression analyses (*PvDREB1F*, *PvDREB2A*, *PvDREB5A*, and *PvDREB6B*) using RT-qPCR in common bean genotypes of different backgrounds: Mesoamerican (derived from crosses among genotypes originated from areas spanning Central America and Colombia) and Andean (derived from genetic materials from the Andes). The first experiment consisted on a temporal (five time periods) and spatial (roots, stem, and leaves) analysis of the *PvDREB* transcripts, using the common bean genotype BAT 477 submitted to abiotic stresses. In the second experiment, besides BAT 477, four other genotypes (BAT 93, Jalo EEP558, IAC-Carioca 80SH, and RAB 96) were included. A spatial (roots, stem, and leaves) analysis of relative gene expression was performed under the same stress treatments, but with only one time period of stress induction.

The genotype BAT 477 has been used in several studies aimed at screening drought performance. BAT 477 has a Mesoamerican background and was developed at the Centro Internacional de Agricultura Tropical (CIAT, Colombia), being used as the drought-tolerant parental line of the mapping population BAT477 × DOR364, which showed QTLs associated to drought in common bean [59]. In the first run of experiments (temporal approach), we only used BAT 477, considering its background for studies in stress tolerance (drought, specifically) and that we were aimed at understanding the variation of expression during increased time periods of exposure to stress.

For the first set of experiments, seeds of BAT 477 were surface-sterilized in 10% sodium hypochlorite for 3 min and rinsed 3-4 times (1 min each time) in distilled water. Plants were grown in pots with sand/vermiculite (1 : 1, *v*/*v*) in a growth chamber at 26°C ± 3 (14 h photoperiod, ~60% air moisture, and light intensity of 120 *μ*mol.m^−2^.s^−1^) and were normally watered until the first trifoliolate leaf was completely expanded (after about 21 days, referred to as vegetative 3 (V_3_) stage). After that, whole plants were removed from pots and subjected to four stress treatments: polyethylene glycol (PEG 10%) solution for dehydration stress, NaCl solution (250 mM) for salt stress, and 100 *μ*M abscisic acid (ABA) solution (Supplementary File 5). ABA treatment was included since different reports have shown that *DREB* genes might be ABA-independent or dependent [3, 5], but our study is only aimed at showing the responsiveness to such treatment. Furthermore, three plants were incubated in a cold chamber (4°C). Control treatment consisted in plants placed in distilled water for comparison with PEG, NaCl, and ABA, while they were kept in pots only irrigated at room temperature for comparison with the cold-treated plants. Treatments were applied for different periods of exposure to each abiotic stress-inducive factor (after 5 min, 30 min, 1 h, 3 h, 6 h, and 12 h; see Supplementary File 5 for details on each stress). Right after the period of exposure (time point) for each stress treatment, three plants were collected for the analyses. All treatments were considered as independent experiments. After each treatment, roots, stem, and leaves were separately placed in tubes properly identified and kept in liquid nitrogen until being transferred to an ultrafreezer (-80°C).

In the second set of experiments, seeds from the Mesoamerican genotypes BAT 477, BAT 93, IAC-Carioca 80SH, and RAB 96 and the Andean Jalo EEP558 were treated in a similar manner than in the first assay. BAT 93 and Jalo EEP558 are the parental lines from the core mapping population of common bean [60]. IAC-Carioca 80SH is a drought-sensitive or moderately sensitive cultivar [61] as well as the breeding line RAB 96 [62]. The same four treatments (PEG 10%, NaCl 250 mM, low temperature, and ABA 100 *μ*M) were applied to three plants of each genotype. However, all stresses were induced for a three-hour period, intermediate point–selected based on the first experiment. Samples were all collected separately and frozen.

Before sampling, however, in order to give indications that plants were effectively suffering from the imposed stress conditions, we determined the relative water content for all samples. Fully expanded leaves were excised, and fresh weight (FW) was recorded; then, leaves were soaked into deionized water for 4 hours and turgid weight (TW) was recorded. All samples were placed in an air oven at 60°C, and total dry weight (DW) was recorded after 24 h. Relative water content (RWC) was calculated according to Barrs and Weatherley [63]: RWC (%) = [(FW–DW)/(TW–DW)] × 100. As a biochemical indicator, catalase (CAT) activity was determined for each sample. Leaf samples of each treatment were frozen and grinded for analyses. A 100 mg leaf tissue sample was used for protein extraction in phosphate solution pH 7.0 with antioxidants (PVPP). Protein quantification was performed using Bradford reagent (Bio-Rad) and following the procedures of Bradford [64]. For CAT assay, 100 *μ*L of each protein sample were placed in a cuvette with 3 mL of phosphate buffer and 60 *μ*L of H_2_O_2_ 30% solution was added. Absorbance decrease was monitored with a NanoDrop™ 2000c (Thermo Scientific) spectrophotometer for 2 min, with measures at each 10 s. Results were expressed in *μ*.mol.min^−1^ mg^−1^ of protein. RWC and CAT changes over time and among genotypes were statistically evaluated with ANOVA, following basic principles of adherence (normality and variance homogeneities). Significant results were further compared with Tukey's test (*P* < 0.05).

### 2.9. RNA Extraction and Gene Expression Profiling with RT-qPCR

RNA extraction was performed with 100 mg tissue samples using TRIzol® Reagent (Invitrogen™) and following the manufacturer's instructions. Quantification and quality were checked with a NanoDrop™ 2000c (Thermo Scientific) spectrophotometer. Gel electrophoresis (agarose 1% in TAE buffer 1x) was also performed for quality assay. Primers flanking the 150-250 pb length across the coding region, trying to avoid the AP2-coding sequence of the four genes, were designed for RT-qPCR (Supplementary File 4). Two reference genes were used for the reactions, being chosen according to tissue analyzed (*SKIP16* and *IDE* for roots and *UBQ* and *IDE* for stem and leaves) and on gene stability, previously studied [65]. A 100 ng RNA sample of each treatment was used to synthesize the first cDNA strand using the Maxima First Strand cDNA Synthesis kit (Fermentas). Quantitative PCR reactions were prepared using 1 *μ*L of newly synthesized cDNA, 0.25 *μ*M of each primer, and 1x SYBR® Green PCR Master Mix (Thermo Scientific). Amplifications were performed on the StepOnePlus™ Real Time PCR System (Applied Biosystems) equipment with the following steps: 10 min at 95°C, 40 cycles of cDNA amplification at 95°C for 15 s, 59°C for 30 s, and 72°C for 20 s with fluorescence signal recording at this stage. A final step at 95°C for 15 s and at 60°C for 1 min, with fluorescence measurements at each 0.7°C variation (from 60 to 95°C), was included to obtain the melting curve. All reactions were performed in triplicates.

The expression data were analyzed following similar approaches as described by Borges et al. [65]. Raw data with fluorescence levels were submitted to LinRegPCR software [66]. Fluorescence was baseline-corrected, and linear regression analysis was performed for all amplifications. The optimal set of data points (Window-of-Linearity) was defined to allow the calculation of the threshold and quantification cycle (*Cq*). Sample efficiencies were calculated based on the slope of the line, ranging from 1.8 to 2.0 and with correlation of at least 0.995. Relative expression data were obtained by REST software [1] using average values of efficiency and *Cq* of target and reference genes. This software calculates the concentration of expression (*C*) by comparing control and treated *Cq* values and determines the relative expression (RE) ratio: RE = *C*_target gene_/geometric average *C*_reference gene_. After, *P* values are obtained by a pairwise reallocation randomization test (bootstrap = 2,000 permutations).

### 2.10. BARCBean6K_3 Bead Chip Analysis

We also aimed to identify genomic positions within or nearby each one of the putative *DREB* genes, which could be useful for diversity, mapping, and association mapping analyses. Then, we traced SNP markers nearby all 54 putative DREB loci previously identified, using the SNP position of the BARCBean6K_3 BeadChip, a SNP array developed for common bean which comprises 5,398 markers distributed along the 11 chromosomes and some nonaligned scaffolds [47]. Since the chromosomal positions of all SNP from the array are known, the nearest SNP to the transcription initiation site of each DREB was searched.

We used the SNP chip to analyze the diversity of common bean genotypes considering all SNP and the specific loci identified near all *DREB* genes. In total, 18 genotypes were genotyped, including 11 Mesoamerican and six Andean and one line from *P. acutifolius*, as an outgroup. The Mesoamerican lines BAT 93, BAT 477, IAC-Carioca 80SH, and RAB 96 and the Andean Jalo EEP558 were included (described earlier in expression profiling experiments). Moreover, we extracted DNA from the drought-sensitive Mesoamerican genotype Rosinha G2. Midas (domesticated Andean) and G12873 (wild Mesoamerican) were also included, representing the population used for mapping traits associated with the domestication syndrome [67]. Another accession used was PI311859, of Mesoamerican origin. The third set of parental lines was IAC-Una (Mesoamerican) and CAL 143 (Andean), used to develop a mapping population screened for growth habit in Brazil [68]. Another Mesoamerican line used is SEA-5, a drought-tolerant line developed at Centro Internacional de Agricultura Tropical (CIAT) and used in QTL mapping for drought-related traits [69]. Two other lines were the Mesoamerican ICA Bunsi (white pea bean developed at Instituto Colombiano Agropecuario) and SXB 405 (cream-seeded, drought-tolerant, and high-yielding breeding line from CIAT). Experiments with 78 inbred lines in Ethiopia showed differences in drought tolerance levels based on pod harvest index [70]. Two lines developed at the University of California Davis, the Andean UCD 0801 and UCD Canario 707, were also genotyped. Finally, the Andean G19833 was used as the reference genotype for comparing the sequences, since it is the line used for common bean genome sequencing [46]. Moreover, the tepary bean (*P. acutifolius*) accession G40111 was used as an outgroup for the analysis. Plants of each genotype were grown in pots filled with soil in a greenhouse. Leaf tissue was collected from the first trifoliolate leaf (V3 stage) and lyophilized.

DNA extractions were performed with a modified version of Doyle [58] protocol. DNA samples from the 18 genotypes described were diluted to 100 ng.*μ*L^−1^. Samples were genotyped with the SNP array at the Soybean Genomics and Improvement Laboratory (ARS/USDA) in Beltsville, Maryland. Intensity data were processed using Genome Studio software v.2011.1 (Illumina Inc., San Diego, CA, USA). Allele calls were performed with a no-call threshold of 0.15 with posterior clustering refining using heterozygotes of reference. Multivariate analysis using principal coordinate analysis (PCoA) was performed with genotypic data, based on a distance matrix, calculated with the Microsoft Excel macro GenAlEx 6.5 [71].

### 2.11. Sanger Sequencing of Specific *DREB* Genes for Nucleotide Diversity Analysis

Partial sequences of the four genes (*PvDREB1F*, *PvDREB2A*, *PvDREB5A*, and *PvDREB6B*) cloned in this study were obtained in the same set of genotypes that was evaluated with the BARCBean6K_3 BeadChip. DNA from all genotypes was diluted to 20 ng *μ*L^−1^. Each PCR reaction was prepared to a final volume of 50 *μ*L for further purification of amplification products. Reactions contained 1x reaction buffer (2 mM Tris-HCl pH 8.4, 5 mM KCl), 3 mM MgCl_2_, 0.2 mM dNTP, 0.2 *μ*M of each primer, and 1 U of High Fidelity Taq DNA Polymerase (Invitrogen). All primers designed for amplifying the *PvDREB* genes were set for annealing temperature at 59°C (Supplementary File 4). Amplification conditions were set as follows: 94°C for 2 min for initial denaturation, 38 cycles of denaturation (94°C for 30 s), primer annealing (59°C for 30 s) and extension (68°C for 1 min), plus seven extra minutes for final extension at 68°C. All reactions were checked in 1.2% agarose gels for unique fragments at the expected size.

Amplification products were purified using the Wizard SV Gel and PCR Clean-Up System (Promega), following the instructions of the manufacturer. Purified samples were quantified and prepared for sequencing in both directions (forward and reverse) using the same pair of primers from the original amplifications. Sequencing was performed at the UC DNA sequencing facility (University of California, Davis), with the ABI 3730 Capillary Electrophoresis Genetic Analyzer using the ABI BigDye Terminator v. 3.1. Cycle Sequencing kit.

All sequences were submitted to quality analysis with DNA Baser version 4.20.0.36 (Heracle BioSoft). Contig assembly was performed with forward and reverse sequences. Only sequences with a quality value higher than 20 were considered for the next steps. High-quality contigs were aligned with BioEdit Sequence Alignment Editor with the ClustalW multiple alignment algorithm. Alignment was also performed with CLC Sequence Viewer version 7.6 (QIAGEN Aarhus A/S), for double-checking. Alignments were used to analyze the presence of SNP among the genotypes. Polymorphic information content (PIC) of each SNP was calculated according to Nayak et al. [72], with the equation PIC = 1−∑(1 − *p*_*i*_^2^), where *p*_*i*_ stands for the frequency of the *i*th allele. The number of haplotypes was determined with DnaSP [73].

DNA sequences were translated into proteins in CLC Sequence viewer version 7.6, and proteins were aligned with the ClustalW algorithm. Nonsynonymous substitutions were checked with the alignments using DnaSP.

## 3. Results

### 3.1. DREB Proteins Were Categorized according to Five Criteria

Searching the common bean sequences available on Phytozome, we initially found 184 unigenes containing at least one AP2 domain (Supplementary File 1). After annotation on NCBI/BLAST and Pfam, three sequences contained one AP2 and one B3 domain (Phvul.003G111800.1, Phvul.007G102800.1, and Phvul.007G002900.1), which fits the basic feature of a RAV protein [74]. Other 20 sequences contained between two and four AP2 domains, similar to AP2 proteins [75]. The sequence Phvul.001G131300.1 revealed an AP2 superfamily domain and was annotated as an AP2-like ERF in soybean (*e* − value = 0). The remaining 157 sequences had only one AP2 domain. These 157 putative *ERF* genes were distributed among the 11 chromosomes of common bean. Their annotation with an *e*-value cutoff of 1 × 10^−5^ provided an initial assessment of the putative *DREB* gene sequences in the common bean genome, but further phylogenetic analyses strengthened the categorization.

The phylogenetic analysis encompassed common bean protein sequences, complemented with 147 sequences from *A. thaliana* and 359 sequences from *G. max* and sequences from NCBI, GenBank, and *AtDREB* genes deposited for *A. thaliana* on TAIR. The neighbor-joining algorithm was applied to grouping all sequences and generating phylogenetic trees. Overall, the analysis demonstrated high homology between two soybean sequences for each one of common bean, consistent with the duplication event of the allotetraploid *G. max* after divergence of *P. vulgaris* [53]. In general, one or more sequences from *A. thaliana* were positioned in the same clade as orthologs from soybean and common bean (Supplementary File 2).

In general, sequences from *A. thaliana* fitted their original categorization from A-1 to A-6 subgroups of *AtDREB* [2], enabling subgroup division for the common bean sequences (data not shown). *AtDREB1A*, *AtDREB1B*, and *AtDREB1C* were in the same clade in group A-1. *AtDREB2A*, *AtDREB2B*, and *AtDREB2C* were in the same group as the previous isolated genes *GmDREBa* and *GmDREBc* [19]. A sequence from *P. acutifolius* predicted as *DREB2C*-like was also included in the same group. The only member of the A-3 group in *Arabidopsis* (*AtABI3*) was grouped with two genes from *G. max* and only one from common bean (Phvul.008G222400). Members of group A-3 were closely related to A-2 [5]. *AtTINY* (A-4) was in the same group as *GmTINY*. *AtRAP2.9*, *AtRAP2.10*, and *AtRAP2.1* were in the same group as *GmDREB2*, all from the A-5 group. Genes *AtRAP2.4* and *AtRAP2.4B* were also in the same clade (A-6) and the soybean gene *GmDREBb* fitted in the same phylogenetic group. All common bean sequences within each of the phylogenetic groups (determined based on *Arabidopsis* and soybean) were considered as putative candidates for the *DREB* gene subfamily, giving in total 57 sequences (Supplementary File 2).

**Figure 1:**
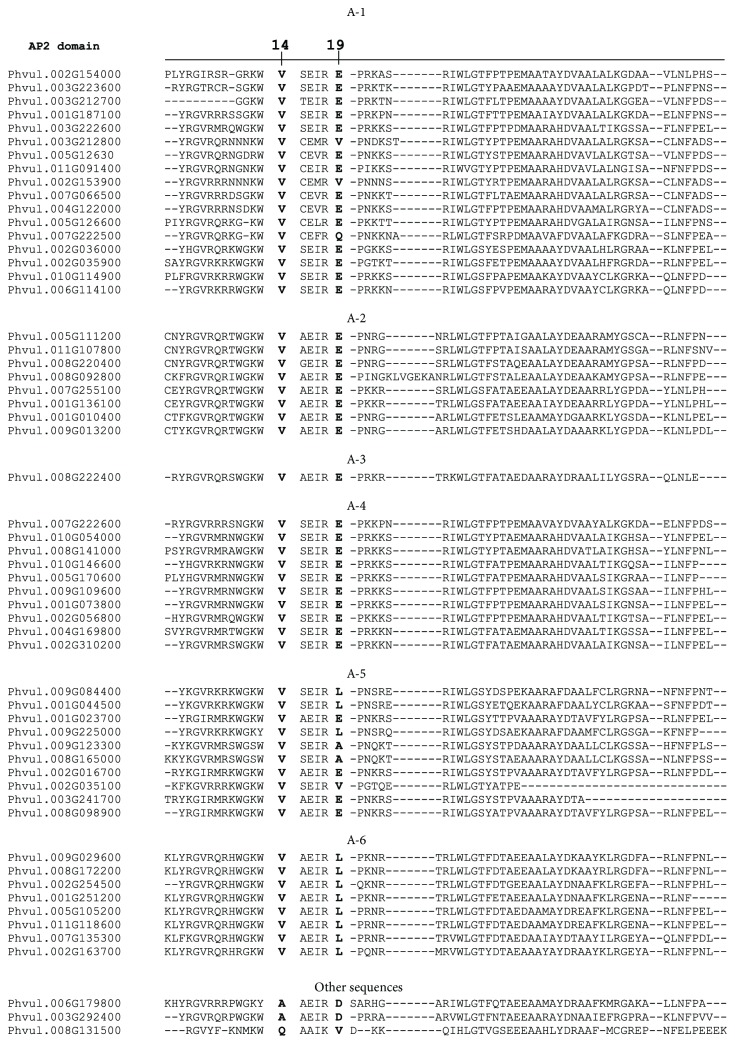
Alignment of the AP2 domain of 54 putative DREB proteins from common bean. Position 14^th^ and 19^th^, described as important for protein binding, are separated by spaces along the sequences. Position 14^th^ presents 100% conservation of the amino acid valine (V), while the 19^th^ varies, although glutamic acid (E) and leucine (L) are the most frequent. Other sequences than the 54 DREB were compared in the alignment. Those sequences fitted as putative DREB in the phylogenetic analysis, but not for amino acid conservation.

Another criterion to define the putative DREB proteins was the amino acid conservation along the AP2 domain. Previous work demonstrated that *DREB* genes have positions 14^th^ and 19^th^ conserved, respectively, with valine and glutamic acid [6]. However, the 19^th^ amino acid might have some variability among proteins. We extracted the AP2 domain sequence from all sequences on NCBI Domain Finder and performed ClustalW global alignment with the 57 sequences categorized from the phylogenetic tree (Figure 1). Fifty-four sequences presented the amino acid valine at the 14^th^ amino acid of the AP2 domain, while the 19^th^ site was represented by glutamic acid in all A-2, A-3, and A-4 members and 14 proteins from the A-1 subgroup. Two peptide sequences from the A-1 subgroup presented valine at this site (Phvul.003G212800 and Phvul002G153900), whereas one sequence had glutamine (Phvul.007G222500). Valine has been pointed out as the most important amino acid for binding affinity (Sakuma et al. [6]). The other three sequences (Phvul.006G179800, Phvul.003G292400, and Phvul.008G131500) had different amino acids at the 14^th^ site (alanine and glutamine) and uncommon amino acids for DREB at the 19^th^ site (aspartic acid and valine), and therefore, they were excluded from the list of DREB proteins (Figure 1, Other sequences). In fact, alanine and aspartic acids are typical from ERF sequences [6].

**Figure 2:**
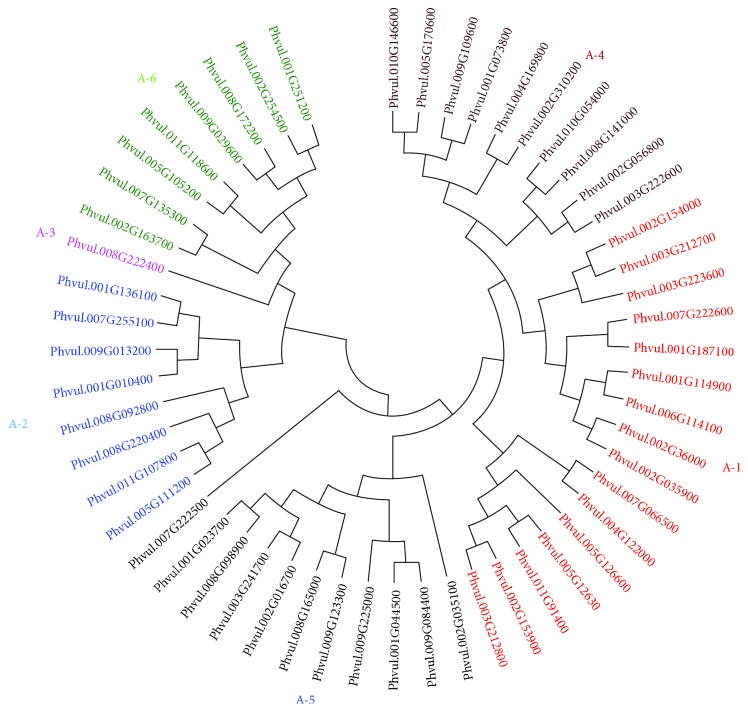
Neighbor-joining tree of 54 putative PvDREB protein sequences. Sequences were retrieved from the common bean (*Phaseolus vulgaris*) genome database on Phytozome. Subgroups of DREB proteins are shown in different colors. Phvul.007G222500 was categorized as an ERF protein and was used as an outlier.

From our analyses, 54 putative DREB proteins were categorized, fitting subgroups A-1 to A-6, according to previous analyses with *A. thaliana* [5] and *G. max* [21].  Figure 2 shows the final neighbor-joining phylogenetic tree with the putative PvDREB proteins. Subgroups A-1 and A-4 were phylogenetically more similar as early reports suggested for other species [5, 6]. Each group presented a different number of proteins (A − 1 = 17, A − 2 = 8, A − 3 = 1, A − 4 = 10, A − 5 = 10, A − 6 = 8).

### 3.2. Protein Motifs Indicated Differences among the Subgroups of the DREB Subfamily

**Figure 3:**
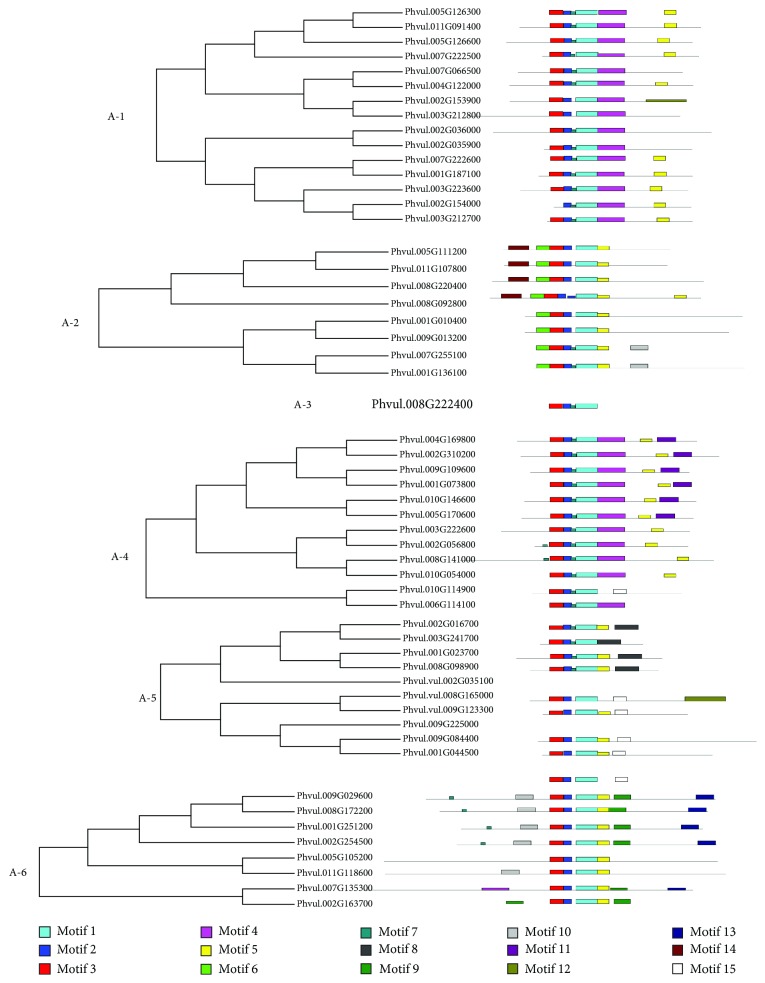
Conserved motifs (motif 1 to motif 15, color-coded) along the amino acid chain of 54 putative PvDREB protein sequences divided by subgroups (A-1 to A-6), showing within-group dendrograms of each subgroup. The conserved motif 15 is represented; however, it showed significant similarity with motif 4. Therefore, 14 unique motifs have been identified.

After categorization and subgroup division, a protein motif prediction search was performed with MEME Suite for the 54 putative DREB proteins. In this tool, users define the number of motifs to be searched against databases. Our criterion to define the maximum number of motifs was based on determining the number before there were no similarities or redundancies among motifs in the list. We determined 14 motifs (1 to 14, described as conserved motifs (CM)) (Supplementary File 6), represented in Figure 3. A 15^th^ motif is also represented, but it presented high similarity with motif CM4, not being considered for comparisons.

Motifs CM1 (RIWLGTFPTPEMAARAYDVAAYCLKG), CM2 (WGKWVCEIR), CM3 (GGPENRHCVYRGVRQR), and CM7 (EPRKK) were found within the AP2 domain (Figure 3). All sequences had CM1, CM2, and CM3. On the other hand, CM7 was detected in all DREB subgroups, but not all sequences. In the A-6 subgroup, CM7 was observed in four sequences (Phvul.009G029600, Phvul.008G172200, Phvul.001G251200, and Phvul.002G254500) closer to the N terminus position.

Some motifs were exclusive to specific DREB subgroups (Figure 3). Motif CM6 (KKVPAKGWKKGCMRGK) was unique to all sequences from the A-2 subgroup. Motif CM8 (DMSADSIRKKATQVGARVDALQTALHHH) was only encountered in four sequences (Phvul.002G016700, Phvul.003G241700, Phvul.001g023700, and Phvul.008G098900) of the A-5 subgroup. Another example is motif CM13 (YWEDDSDHFNLQKYPSYEIDW), only detected in five DREB proteins (Phvul.009G029600, Phvul.008G172200, Phvul.001G251200, Phvul.002G254500, and Phvul.007G135300) from the A-6 subgroup. Motif CM10 (LNHLTPPQVHQIQAQIQIQKQ) was only detected within A-6 sequences as well. Motif 14 (HSKGDGSKSVADTLAKWKEYNAQL) was found in A-2 and A-4 subgroups, but in different positions along the peptide sequence (near N-terminus in A-2 and near C-terminus in A-4).

Exclusive motifs defined phylogenetic markers identifying DREB subgroups. They might have specific functional roles for each one of the genes. Therefore, motif identification and categorization in this work are important for further steps aimed at the molecular and functional characterization of *DREB* genes of common bean.

### 3.3. Chromosomal Distribution of the *PvDREB* Genes

**Figure 4:**
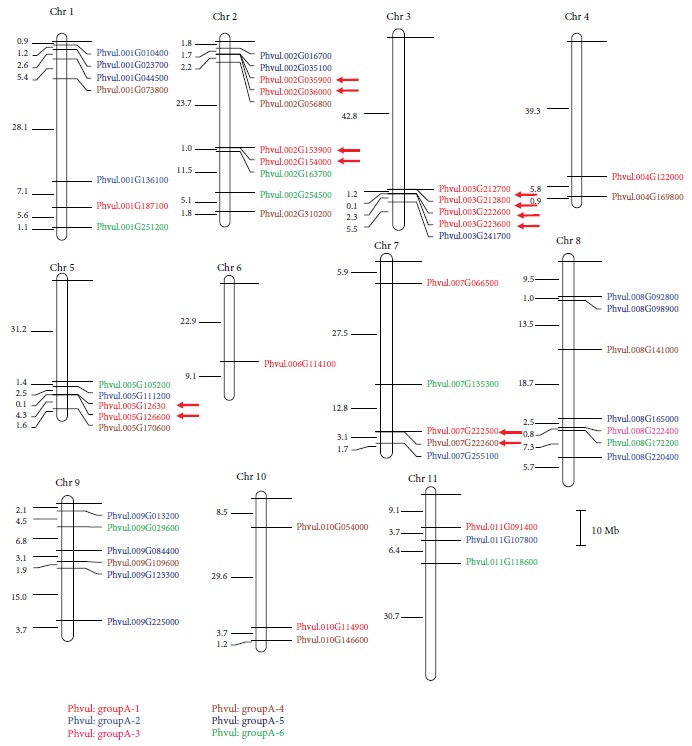
Chromosomal location of 54 putative *PvDREB* genes. Subgroups are represented by different colors. Red arrows indicate possible recent tandem duplication events.

The chromosomal location of all the putative 54 *PvDREB* genes is represented on the map in Figure 4. Gene distribution along chromosomes is not separated by the *DREB* subgroup (A-1 to A-6), although some groups of genes within the same subgroup were observed such as members of A-1 on chromosomes 2, 3, and 5. Six pairs of genes indicate to be tandemly duplicated (Figure 4) since they are located within a distance around 100 kb or less and are not separated from more than five genes (Phvul.002G035900 and Phvul.002G036000, Phvul.002G153900 and Phvul.002G154000, Phvul.003G212700 and Phvul.003G212800, Phvul.003G222600 and Phvul.003G223600, Phvul.005G126300 and Phvul.005G126000, and Phvul.007G222500 and Phvul.G222600) (Figure 4 and Supplementary File 7). In general, these possible duplications happened with genes from the A-1 subgroup, with one exception between one A-1 and one A-4 genes (Phvul.007G222500 and Phvul.G222600, respectively) (Figure 4). The proximity of Phvul.002G035100 with Phvul.002G035900 and Phvul.002G036000 is also an indication of genes derived from one of them. Supplementary File 7 shows the distances between pairs of genes, considering all putative *DREB* genes. The criterion adopted maybe too strict (chromosomal proximity and similarity) to define duplications, since it might represent more recent events. Other duplications might have happened in previous events, but chromosomal rearrangements and mutations might have increased the differences among genes.

### 3.4. Gene Ontology Analysis

**Figure 5:**
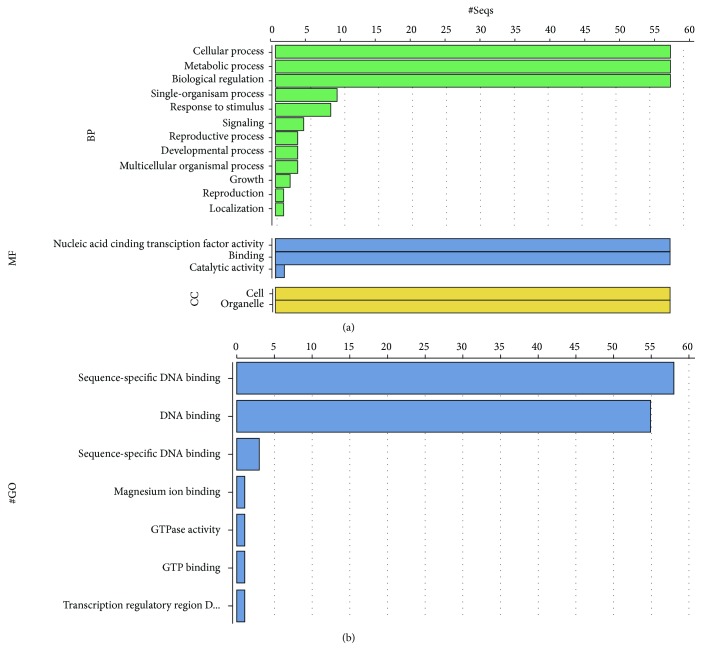
Blast2Go annotation of the putative PvDREB proteins. (a) Go distribution by level—processes. (b) Direct GO count. BP: biological process; MF: molecular function; CC: cellular component.

The gene ontology analysis on Blast2Go suggested that all sequences are involved in sequence-specific DNA binding, the basic characteristic of transcription factors. Furthermore, predictions showed all proteins are localized to the nucleus (Supplementary File 8).  Figure 5 shows all predictions obtained for basic processes, molecular functions, and the GO terms of the putative PvDREB protein sequences.

### 3.5. The Genes *PvDREB1F*, *PvDREB2A*, *PvDREB5A*, and *PvDREB6B* and Their Nomenclature

**Figure 6:**
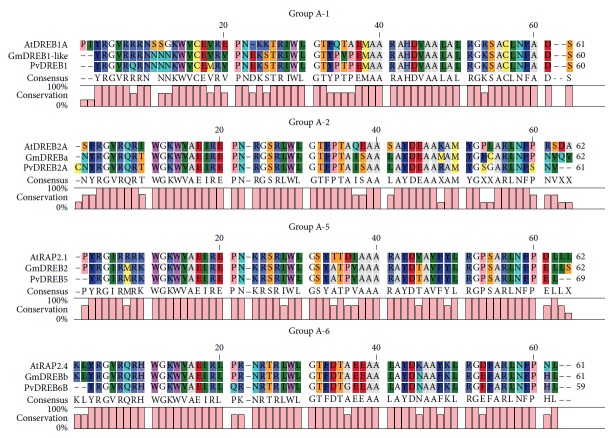
AP2 domain alignment and conservation among the proteins PvDREB1F, PvDREB2A, PvDREB5A, and PvDREB6B and homologs from *Arabidopsis thaliana* and *Glycine max*.

An in silico analysis using Genevestigator platform (https://genevestigator.com/gv/) allowed verifying the expression profile of the main *A. thaliana DREB* genes under several abiotic stresses (Supplementary File 3). We used this information to search the ortholog genes in common bean and initiate studies on their gene expression profile under selective treatments. We cloned four *DREB* genes from common bean and named them after their expression patterns as well as phylogenetic relationships with *A. thaliana* and soybean genes. *PvDREB1F* (GenBank KX151399, or in Phytozome Phvul.003G212800.1) has high homology with the *A. thaliana* genes AT1G12610.1 (*AtDREB1F/DDF1*) and AT1G63030.1 (*AtDREB1E/DDF2*), from subgroup A-1. *PvDREB2A* (GenBank KX151398, Phvul.011G107800.1) is homologous to *GmDREBa* (A-2 subgroup) from soybean and to the *A. thaliana DREB2* genes. *PvDREB5A* (GenBank KX151397, Phvul.008G098900.1) is homologous to *GmDREB2* (A-5 subgroup) and to the *A. thaliana RAP2.1*, from the A-5 subgroup. *PvDREB6B* (GenBank KX147642, Phvul.002G254500.1) is homologous to *GmDREBb* (A-6) and to the *A. thaliana* genes AT2G22200.1, AT4G39780.1, and AT5G65130.1 (all from A-6).  Figure 6 shows the alignment and conservation of the AP2 domain of the four DREB from common bean with homolog proteins from *A. thaliana* and *G. max*.

*PvDREB6B* has been found to be equivalent to the *PvDREB2A* of previous studies [72, 76]. At the time, the genomic sequence of common bean was not available and only a few sequences were deposited on GenBank (NCBI), which resulted in limited annotation precision. The current version of the genome shows the complete genomic sequence for this gene, and its phylogenetic analysis clearly suggests its homology with A-6 genes. In addition, the annotation suggests it is similar to a *RAP2.4* gene from *A. thaliana*, one of the most studied members of the A-6 subgroup. In Supplementary File 9, we show a phylogenetic tree with the sequence used by Nayak et al. [72] and Cortés et al. [76] and from the current study. Here, we proposed the replacement of the name for *PvDREB6B* (deposited to the GenBank as KX147642.1).

### 3.6. Phyto Mine Expression Profile

**Figure 7:**
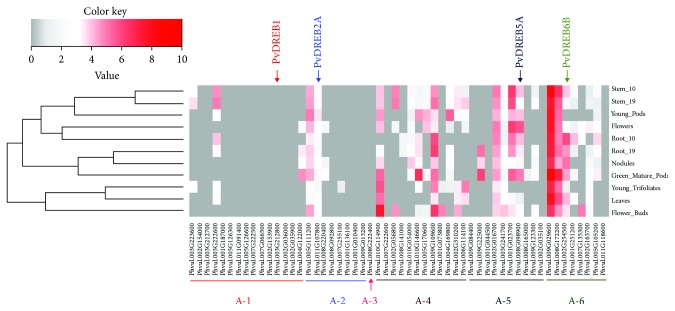
Heatmap of converted FPKM values retrieved from Phytozome database (RNA-Seq data) for 54 putative *PvDREB* genes. The genes *PvDREB1F*, *PvDREB2A*, *PvDREB5A*, and *PvDREB6B* are indicated.

After determining all the putative *DREB* genes in common bean, we compiled data from RNA-Seq analysis deposited on Phytozome to verify the basal levels of expression of each gene in several plant tissues, using FPKM (Fragments per Kilobase of Exon per Million Fragments Mapped) values. The FPKM values suggested different basal levels of transcripts in the tissues of common bean (Figure 7). In general, most A-1 and A-2 genes had very low levels (FPKM value ≤ 1). *PvDREB1F* showed negative FPKM values, which were converted to zero (transcripts nearly undetectable) in all tissues. *PvDRE2A* had considerable transcript amounts in all tissues (mean FPKM = 2.79). The only member of the A-3 subgroup (Phvul.008G222400) had negative FPKM in all tissues. Higher amounts of transcripts were detected in most A-4, A-5, and A-6 genes. *PvDREB5A* and *PvDREB6B* had high positive values of FPKM (means FPKM of 3.21 and 3.45, respectively) (Figure 7).

### 3.7. Identification of SNP Nearby the Entire *DREB* Gene Subfamily and Their Genotyping

The closest SNP marker to each of the 54 previously categorized *PvDREB* was identified through the BARCBean6K_3 BeadChip, a SNP array developed for common bean (Supplementary File 10). Based on their chromosomal location, the distance between the transcription initiation site to the most proximal SNP from the chip ranged from 526 bp (Phvul.002G016700 to SNP ss715639434) to 362,854 bp (Phvul.010G146600 to SNP ss715645496). In fact, SNP ss715639434 was the only located within a distance less than 1,000 bp from a *PvDREB* gene (Phvul.002G016700, from the A-5 subgroup). Several SNPs were identified within a 10 kb distance from the initiation site of *PvDREB* genes: Phvul.001G010400 (4,378 bp), Phvul.001G073800 (9,353 bp), Phvul.001G187100 (3,074 bp), Phvul.002G036000 (8,525 bp), Phvul.002G056800 (2,638 bp), Phvul.003G212700 (5,786 bp), Phvul.003G212800 (1439 bp), Phvul.005G105200 (8,451 bp), Phvul.G126300 (7,091 bp), Phvul.005G170600 (5,533 bp), Phvul.007G255100 (5,819 bp), Phvul.008G098900 (8,262 bp), Phvul.008G165000 (8,380 bp), and Phvul.010G114900 (3,285 bp) (Supplementary File 10).

The nearest SNP to each of the four genes studied in this work (*PvDREB1F*, *PvDREB2A*, *PvDREB5A*, and *PvDREB6B*) were also identified in the platform (Supplementary File 10). SNP ss715645943 was only 1,439 bp apart from the initiation site of *PvDREB1F* (Phvul.003G212800). SNP ss715639652 was located 20,886 bp apart from the initiation site of *PvDREB2A* (Phvul.011G107800). SNP ss715651042 was the closest marker to *PvDREB5A* (Phvul.008G098900), with a distance of 8,262 bp. SNP ss715649110 was the closest to *PvDREB6B* (Phvul.002G254500), with a distance of 10,194 bp.

Three SNPs from the array were the closest markers to pairs of genes. SNP ss715649534 (chromosome 2) was the closest marker to both Phvul.002G153900 (11,816 bp) and Phvul.002G154000 (55,688 bp). SNP ss715647663 (chromosome 2) was nearby Phvul.002G035900 (24,621 bp) and Phvul.002G036000 (8,525 bp). SNP ss715646516 (chromosome 7) was located nearby Phvul.007G222500 (26,917 bp) and Phvul.007G222600 (34,056 bp). As a result, 51 nonredundant SNP markers were located as potential *DREB*-associated loci.

**Figure 8:**
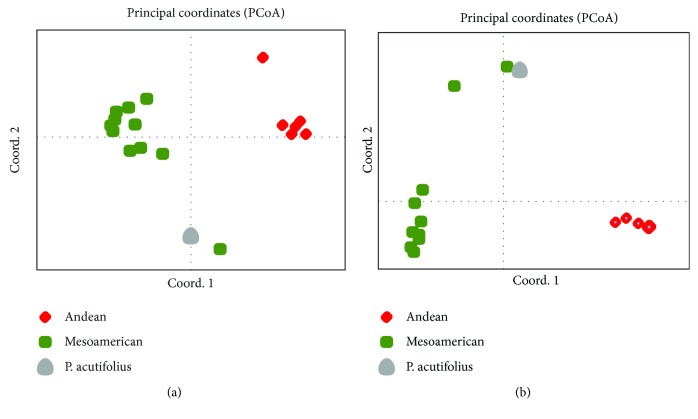
Principal coordinate analysis (PCoA) plot of the genetic structure of 17 common bean genotypes from Andean (G19833, Jalo EEP558, Midas, UCD-0801, UCD-Canario 707, CAL 143) and Mesoamerican (G12873, PI311859, BAT 93, BAT 477, IAC-Carioca 80SH, RAB 96, Rosinha G2, IAC-Una, SEA 4, SxB 405, and ICA Bunsi) background based on (a) 43 SNP markers nearby the initiation site of *PvDREB* genes and (b) 2,995 high-quality SNP calls from the entire BARCBean6k_3 SNP array. An outlier from *P. acutifolius* was added (line G40111).

The SNP array was used to genotype 18 bean genotypes with contrasting origin. Among them, 11 genotypes of Mesoamerican origin, six Andean, and one line from *P. acutifolius* were used. After analysis, 43 high-quality SNP calls of the 51 *DREB*-linked loci were detected in all genotypes. From the analysis of the potential 43 *DREB*-associated SNP markers in the 18 genotypes, a clear separation between Andean and Mesoamerican genotypes was revealed, as shown by principal coordinate analysis (Figure 8(a)). The wild G12873, however, was separated from the other Mesoamerican and close to G40111 (*P. acutifolius*). Similar results were obtained when 2,995 high-quality SNP cells (with no missing data among all genotypes) from the entire chip were used for the analysis (Figure 8(b)). PCoA showed G12873 and PI311859 separated from the domesticated Mesoamerican lines. Thereby, the analysis of the 43 markers showed consistency in determining the basic panorama of the genetic structure of common bean genotypes, as has been shown for whole-genome marker studies and sequence analysis of specific genes. These markers might be useful to the identification of QTL related to abiotic stress responses in common bean populations.

### 3.8. Temporal and Spatial Expression Profiling of the Four *PvDREB* Transcripts

The genes isolated were investigated for their expression profiles under abiotic stress treatments in two experiments. First, we analyzed their expression under a temporal (five periods of stress) and spatial (roots, stem, and leaves) approach, with the following treatments: dehydration (PEG 10%), high salinity (NaCl 250 mM), low-temperature (4°C), and abscisic acid treatment (ABA 100 *μ*M), using a drought-adapted genotype, BAT 477. To analyze morphophysiological changes in plants after stress induction, we measured the leaf relative water content (RWC) and catalase enzyme activity (CAT) for all treatments and periods of stress. Significant changes (*P* < 0.05) were observed from the control to the treated samples, as shown in Supplementary File 11, giving indications of stress at the morphologic, physiologic, and biochemical levels. RWC was significantly changed (*P* < 0.05) with PEG and salinity treatments. CAT activity was altered (*P* < 0.05) with the four treatments, with distinct profiles per treatment.

**Figure 9:**
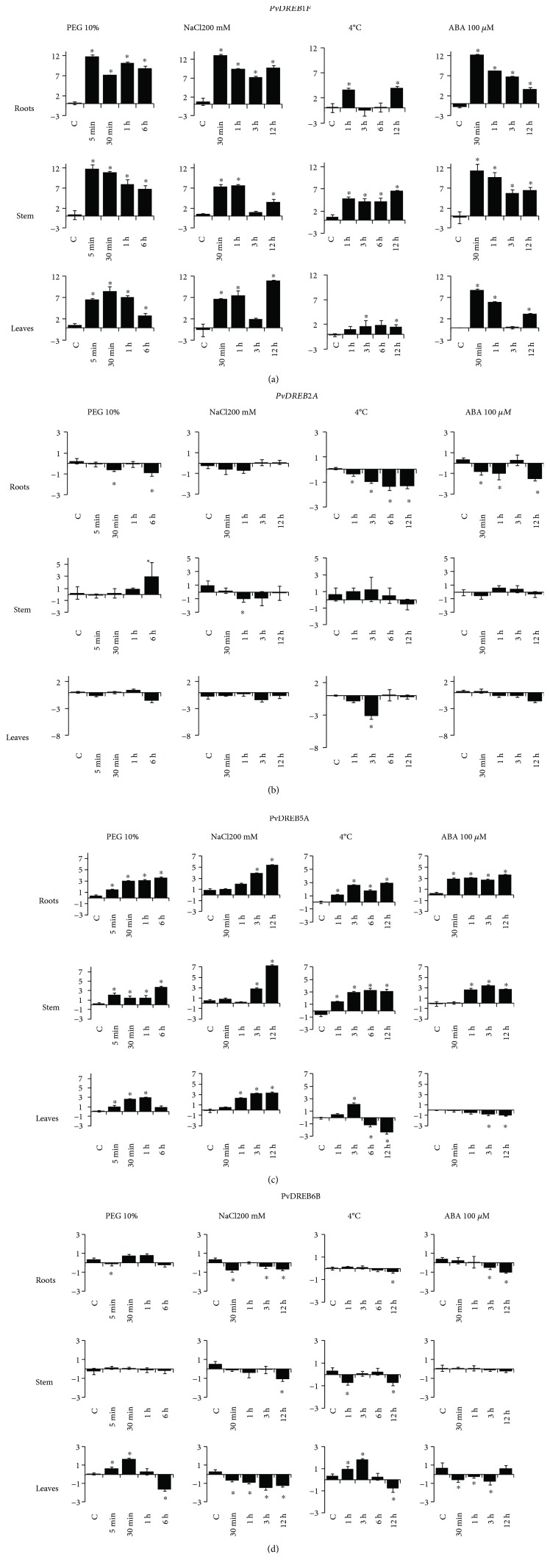
Temporal and spatial scale qRT-PCR gene expression profile of four common bean *DREB* genes: a- *PvDREB1F*, b- *PvDREB2A*, c- *PvDREB5A*, and d- *PvDREB6B*, in BAT 477 (drought-tolerant genotype) plants subjected to different abiotic stress induction: dehydration by using polyethylene glycol (PEG 10%), high salinity by a solution of NaCl 200 mM, cold by incubation at 4°C, and abscisic acid induction factor (ABA 100 *μ*M solution). Values are expressed in relative terms: expression value of stressed samples is relative to control samples. ^∗^ indicates significant up or downregulation of the genes in comparison to their control samples.

Genes *PvDREB1F*, *PvDREB2A*, *PvDREB5A*, and *PvDREB6B* exhibited different patterns of expression under the four treatments (Figure 9). The expression profiles varied according to the period of stress and the plant organ. Transcripts of *PvDREB1F* rapidly accumulated under dehydration (up to 12-fold change in log_2_ units), high salinity (up to 12-fold change), and ABA treatment (up to 12-fold change) in all plant organs (roots, stem, and leaves), in comparison to the untreated plants (Figure 9). In general, relative expression values were lower with the freezing treatment (up to 6.5-fold change) than with the others. Increased expression has been observed in roots after one hour, but lower levels were observed after three and six hours with a final increase after 12 hours of exposition to cold.

*PvDREB2A* had low inducibility under the abiotic stresses of the study, with exception for the dehydration treatment (relative expression three folds higher than control) on the stem of BAT 477 (Figure 9). Some slight increase in the relative number of transcripts was also observed with the cold treatment (up to 1.25-fold change). ABA treatment caused some variation, first with some decrease (until -1-fold change) followed by an increase (to 0.3-fold change) in the relative number of transcripts.

*PvDREB5A* also revealed to be stress-inducible under all treatments. In general, dehydration led to increased inducibility over time in roots (3.6-fold change, 6 h), stem (3.7-fold change, 6 h), and leaves (3-fold change, 1 h) (Figure 9). The same was observed with the treatment with high salinity, with the highest relative expression values after 12 hours of treatment (5.5, 7.3 and 3.3, respectively, for roots, stem, and leaves). Treatment with cold also increased transcript accumulation in roots (2.9-fold change) and stem (3.3-fold change) when compared to the control plants at room temperature. An increase in expression was observed in leaves as well (up to 2.1, 1 h), but it was followed by a high decrease by the time points of six (-1.2-fold change) and 12 hours (-2.4-fold change) of stress. ABA mostly led to an increase in the levels of transcripts in roots (3.7-fold change, 12 h) and stem (3.4-fold change, 3 h), but a slight and progressive decrease was observed in leaves (up to -1.1-fold change).

The most significant aspects about the *PvDREB6B* expression profile were an increase in its levels after treatment with dehydration in roots (up to 0.8-fold change) and leaves (maximum of 1.7-fold change) (Figure 9). Cold treatment led to pronounced expression of *PvDREB6B* in leaves, with a progressive increase (up to 1.8-fold change, 1 h) followed by a decrease (-0.8-fold change, 12 h). Salinity diminished the levels of transcripts in all organs. ABA produced a similar effect, although no significant differences were observed in the stem and some increase in the transcript's relative level was detected after 12 hours of exposition.

### 3.9. Spatial Expression Profiling in Different Common Bean Genotypes

In the second experiment, the same four treatments were applied to five genotypes (BAT 93, Jalo EEP558, BAT 477, IAC-Carioca 80SH, and RAB 96) contrasting for abiotic stress tolerance. Once again, the treatments were applied to elicit different physiological and biochemical responses of each genotype, as evaluated with the relative water content (RWC) and the ROS-scavenging enzyme catalase (Supplementary File 12). However, only one period of stress was applied, three hours of stress, and compared among all genotypes.

**Figure 10:**
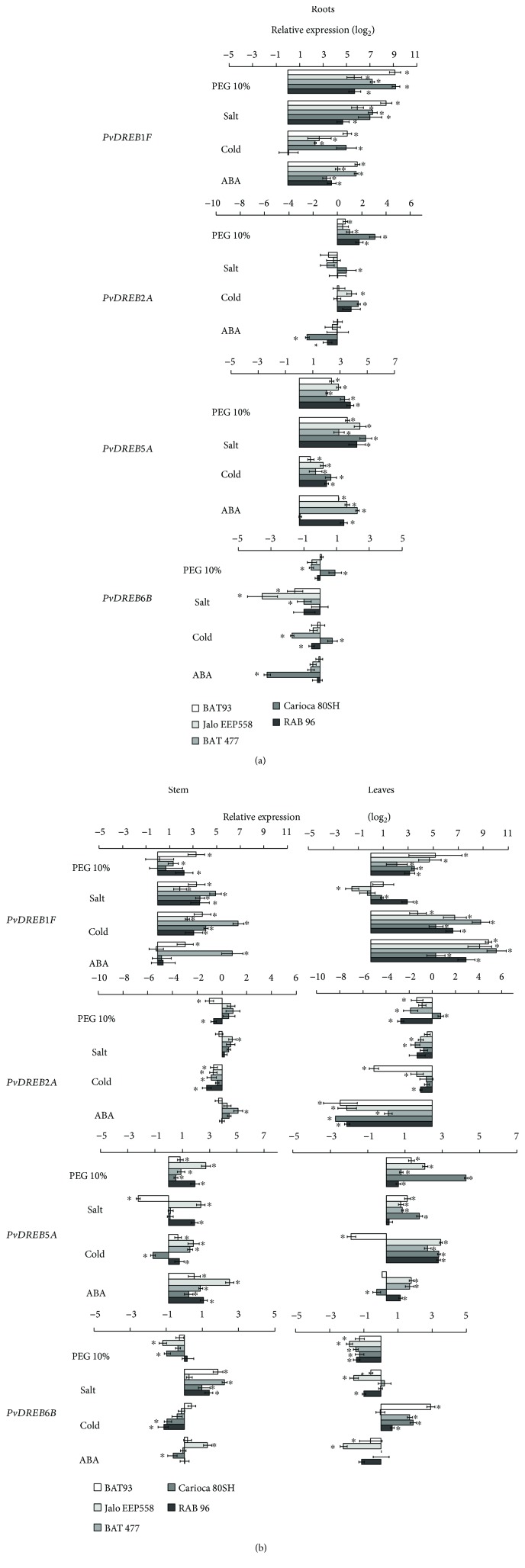
Spatial scale qRT-PCR gene expression profile of four common bean *DREB* genes (*PvDREB1F*, *PvDREB2A*, *PvDREB5A*, and *PvDREB6B*) in BAT 93, Jalo EEP558, BAT 477, IAC-Carioca 80SH, and RAB 96 plants subjected to different abiotic stress induction: dehydration by using polyethylene glycol (PEG 10%), high salinity by a solution of NaCl 200 mM, cold by incubation at 4°C, and abscisic acid induction factor (ABA 100 *μ*M solution). (a) Expression in roots and (b) expression in stem and leaves. ^∗^ indicates significant (*P* < 0.05) up or downregulation of the genes in comparison to the control samples.

As in the first experiment, stresses caused similar responses of each one of the four *PvDREB* genes (*PvDREB1F*, *PvDREB2A*, *PvDREB5A*, and *PvDREB6B*), but with some particular differences in each bean genotype. *PvDREB1F* was strongly induced after the three-hour period of stress in all treatments and genotypes, except under salinity in leaves (Figure 10(b)). *PvDREB2A* expressed under dehydration in roots and stem of most genotypes (Figure 10). The highest relative expression value was observed for the genotype IAC-Carioca 80SH in roots (3.1-fold change). It was also the only genotype with increase in the relative transcript levels in leaves (0.7-fold change). Salinity increased the number of transcripts in roots, as well as ABA in the stem. Exposure to cold augmented the expression in Jalo EEP558 (1.2-fold change), IAC-Carioca 80SH (1.7-fold change), and RAB 96 (1.1-fold change). A strong decrease in transcript levels was observed after ABA treatment in all genotypes.

*PvDREB5A* was induced under all treatments and in all genotypes (Figure 10), as it was in the temporal analysis with BAT 477 (Figure 9). Decreased relative expression level was observed in BAT 93 stems after exposure to high salinity (-1.8-fold change). In the same organ, no difference was observed from control and salt treatment in BAT 477 and IAC-Carioca 80SH, while Jalo EEP558 and RAB 96 had high inducibility. Additionally, BAT 93 was the only one to present a decrease in the transcript level after cold treatment in leaves.

*PvDREB6B* transcripts accumulated with salinity treatment after three hours in the stem of all genotypes. As observed in the temporal experiment, inducibility was also detected under low-temperature exposure in leaves (maximum of 2.9-fold change in BAT 93), with the exception of Jalo EEP558 (-0.05-fold change). In roots, dehydration and low temperature increased relative transcript levels in IAC-Carioca 80SH (0.7- and 0.9-fold change, respectively), the opposite of what was observed for the other genotypes (negative values up to -1.7) (Figure 10).

### 3.10. Nucleotide Diversity of the Four *PvDREB* Genes

**Figure 11:**
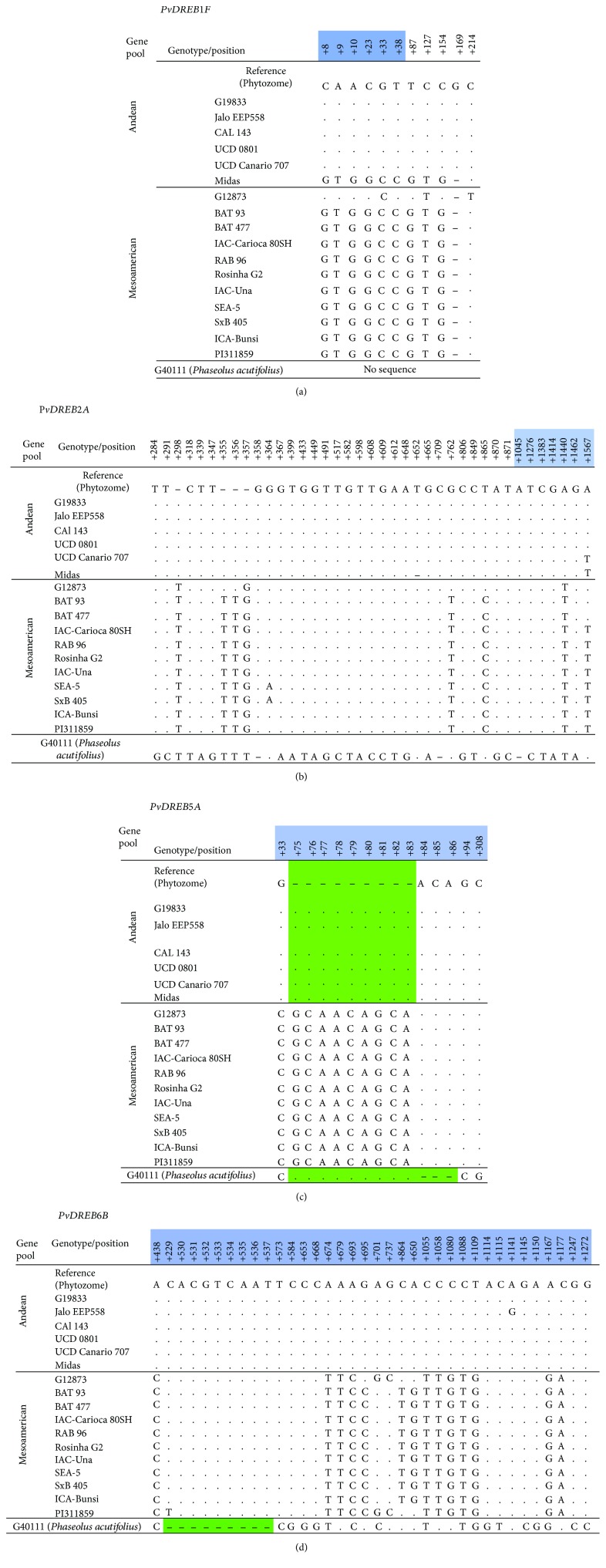
Nucleotide variants profile of four DREB genes from common bean (*PvDREB1F*, *PvDREB2A*, *PvDREB5A*, and *PvDREB6B*) based on their direct resequencing on 17 genotypes with contrasting origin: six Andean and 11 Mesoamerican. An additional line, G40111 (*Phaseolus acutifolius*) was used as an outlier. Spots highlighted in green indicate the location and size of INDEL sites.

The resequencing of *PvDREB1F* (ORF + intron), *PvDREB2A* (ORF + intron 1), *PvDREB5A* (ORF), and *PvDREB6B* (ORF) in 17 common bean genotypes and one *P. acutifolius* line evidenced different numbers of SNP markers and other nucleotide variants within each gene (Figure 11).

The polymorphisms identified within *PvDREB1F* were located in the first exon from the start codon (positions +8, +9, +10, +23, +33, and +38) and the intron between exon 1 and exon 2 (positions +87, +127, +154, +169, and +214) (Figure 11(a)). All the 10 SNPs averaged PIC = 0.432, with five haplotypes (Table 1). *PvDREB2A* exhibited a low number of polymorphic sites within the ORF of 600 bp (Figure 11(b)). Only two SNPs were detected within the common bean panel of genotypes. Additional five polymorphic sites were encountered among G40111 (*P. acutifolius*) and the common bean panel (the sequence obtained for G40111, however, was not complete). Intron 1 from *PvDREB2A* showed seven SNP sites. In general, polymorphisms contrasted genotypes from Andean and Mesoamerican origin. Four SNP (+355, +356, +762, and +865) contrasted the wild G12873 from the other Mesoamerican materials (Figure 11(b)). In average, high polymorphic information content was obtained for all the SNPs (PIC = 0.412, six haplotypes) (Table 1). The lowest number of SNP was detected within the ORF of *PvDREB5A*, a short fragment of 474 bp (reference genotype G19833 with sequence deposited on Phytozome). Only one SNP at position +33 completely distinguished the Mesoamerican from the Andean materials (PIC = 0.475, Table 1). However, an INDEL of 9 bp was encountered between the two gene pools. The short sequence (CGCAACAGCA) was absent in the Andean (ORF = 474 bp) and present in all Mesoamerican genotypes (ORF = 483 bp). The size of the INDEL was higher within G40111, with additional three nucleotides absent in comparison to the Mesoamerican sequences (Figure 11(c)).

The highest number of polymorphic sites was detected within the ORF of *PvDREB6B* (Figure 11(d), Table 1). In total, 18 SNPs were encountered among the common bean genotypes. More 10 SNPs were detected among the *P. acutifolius* line and the common bean materials. Additionally, an INDEL of 9 bp (CACGTCAAT) was detected, being absent within the ORF of G40111 (Figure 11(d)). The high variability of this gene has been previously explored by Nayak et al. [72] and Cortés et al. [76], but at the time those authors were able to construct a contig of only 547 bp. The actual size of the open reading frame of *PvDREB6B* is 957 bp, aided by a 5′-UTR region of 386 bp and a 3′-UTR of 507; in total 1850 nucleotides.

Nucleotide variant sites resulted in nonsynonymous substitutions when the ORFs were translated to protein sequences (*PvDREB2A*, *PvDREB5A*, and *PvDREB6B* only) (Table 1). The point mutation +1440 within *PvDREB2A* resulted in a change from lysine (K) (Andean) to methionine (M) (Mesoamerican). The short INDEL sequence within *PvDREB5A* codes for three units of glutamine (Q). Therefore, while Andean genotypes presented four *Q*s in a row, Mesoamerican had seven units of this amino acid. Finally, the high number of point mutations or frameshifts within *PvDREB6B* resulted in nine nonsynonymous substitutions among the common bean genotypes. The INDEL from G40111 (*P. acutifolius*) represented three amino acids (S, R, and Q) which appeared in common bean but not in G40111.

## 4. Discussion

### 4.1. Phylogenetic Analysis, Motif Predictions, and Expression Profiles

Our work provided a detailed genome-wide categorization of the *DREB* gene subfamily in common bean. In total, 54 putative *DREB* genes were catalogued and divided into six subgroups, according to the previous reports for *A. thaliana* [5, 6]. All proteins represent the common aspects of *DREB* genes, especially the conservation of the 14^th^ and 19^th^ amino acids within the AP2 domain [6]. The number of *DREB* genes categorized for common bean was similar to *A. thaliana*, which has 57 *AtDREB* separated into four main subgroups in the *AP2/ERF* superfamily [5], or 56 within six subgroups, from A-1 to A-6 [6]. In soybean, that number has been described to be much lower, with only 36 putative *GmDREB* [21]. However, the study of Zhang et al. [21] was published before the whole genome sequence of *G. max* was released [53]. Moreover, phylogenetic analyses in the current work show that for several common bean *DREB*, there are two copies in soybean, suggesting a higher number of *DREB* loci in the soybean genome (Supplementary File 2).

After categorizing *PvDREB* genes, we showed putative pairs of genes that might have undergone duplication in their respective chromosomes (Figure 4, Supplementary File 7). Six pairs of genes might represent more recent tandem duplication events. Interestingly, all these six events involved genes from the A-1 subgroup, with one exception involving an A-1 gene and an A-4 gene (Figure 4). A previous report has found an overrepresentation of *DREB1/CBF* genes for *E. grandis*, which could have been an adaptation response to climates where the species were changing over time [30]. It is also well documented that tandem duplications are adaptively relevant to the evolution and function of abiotic and biotic stress-responsive genes. Some experimental evidence revealed that tandem arrays often share regulatory elements and might be coexpressed [77, 78], exhibiting similar functions [79]. An increased representation of *DREB1* genes in common bean might have an adaptive role in a similar manner.

The investigation of protein motifs in all DREB sequences revealed several short conserved regions within DREB subgroups, indicating their potential as phylogenetic markers for each subdivision. The exclusivity of some motifs within subgroups might be related to specific functions in which the protein members are involved. In this work, we have not performed a direct and whole comparison of all motifs with other plant genomes, since our aim was to use such sequences as phylogenetic indicators. However, complimentary analyses have shown motifs shared among the entire set of common bean DREB and some isolated proteins from *A. thaliana* and *G. max* (Supplementary File 13). For example, the alanine-rich motif CM4 was found within members of subgroups A-1 and A-4 from common bean, as well as in *AtDREB1A*, *AtDREB1B*, and *AtDREB1C* (A-1) and *GmTINY* and *AtTINY* (A-4). This indicates many motifs are conserved among species, which could also have a similar function. Moreover, changes in amino acid structure could have shaped their functions across species, details that need further investigation.

Having defined the putative *PvDREB* genes, annotation and gene ontologies suggested all sequences have a DNA-binding ability. However, for common bean, only one *PvDREB* gene has been experimentally tested in this matter (data not published). The present study, then, provides insights for further molecular characterization of *DREB* loci from common bean.

Several transcription factors usually have low basal levels in cells, having their concentration increased when activated by determined stimuli such as abiotic stresses. The FPKM values retrieved from the Phytozome database for all the putative *PvDREB* loci showed very low levels of most A-1 and A-2 *PvDREB* members (Figure 7). These are usually the main regulators towards responding to stresses such as drought, salinity, and cold [3]. The A-3 member also showed very low levels. Most members from the A-4, A-5, and A-6 subgroups exhibited higher levels in all tissues analyzed. Genes from the A-4 subgroup generally show no consistent stress inducibility [13, 14], but they may play a role in configuring stress responses, although the mechanisms are not clear so far [3]. The stress-inducible A-5 members are known by the presence of an ERF-associated amphiphilic repression (EAR) motif [80]. These genes were reported to be upregulated when A-1 and A-2 members were overexpressed [5], and further evidence shows that they act as transcriptional repressors downstream of *DREB1* and *DREB2* genes [81]. A-6 members are also usually stress-responsive, and microarray analyses have shown the main *Arabidopsis* gene, *RAP2.4*, to be involved in the regulation of aquaporins [17]. Therefore, they function in stress regulation, but seem to have different targets than the *DREB1* and *DREB2* genes [3].

### 4.2. Nucleotide Diversity of *PvDREB*-Linked Loci

Searching for molecular markers nearby each one of the putative *DREB*, we localized 51 nonredundant SNP sites proximal to the transcription initiation site of each *PvDREB* genes. Genotyping 17 common bean lines and the outlier from *P. acutifolius*, we verified the potential of these 51 markers to detecting the genetic structure traditionally observed for the species into Mesoamerican and Andean gene pools [46]. Moreover, these results give prospects for further studies aimed at mapping specific traits associated with *DREB*-linked polymorphisms. Potential QTL at the sites where *DREB* genes are located might indicate their contribution to the trait of interest, allowing more efficient selection through such molecular markers.

After Sanger-sequencing partial sequences of the four isolated genes in this work, *PvDREB6B* clearly showed the highest diversity (as expressed by the PIC) and numerous nonsynonymous substitutions. This might have important evolutionary implications and needs further research.

### 4.3. Temporal and Expression Profiles of the *PvDREB* Genes

The expression profiles of the genes *PvDREB1F*, *PvDREB2A*, *PvDREB5A*, and *PvDREB6B* were analyzed under dehydration, salinity, low temperature, and ABA treatments, considering different time periods of stress with the genotype BAT 477. *PvDREB1F* showed the highest relative expression values under all treatments (Figure 9). However, rather low values were observed with the cold treatment, compared to the ones usually expected for some *DREB1* genes, as reported in *Arabidopsis* [3]. This might be explained by the fact that *PvDREB1F* is phylogenetically closer to the genes *DWARF AND DELAYED FLOWERING 1* (*DREB1F/DDF1*) and *DREB1E/DDF2*, which are mainly induced by salinity in *A. thaliana* [82, 83]. Moreover, there is cross-talk between *DREB1* and *DREB2* genes, which might lead to *DREB1* responsiveness to osmotic stresses as well as low temperature [3]. In other species, such as *G. max*, a *DREB1-*like (Glyma10g07770.1) gene was also induced by water deficit [84]. These findings suggest that the stress-responsiveness of such genes has been shaped in different manners among plant species.

Although high relative expression values were found for *PvDREB1F*, a direct comparison with the expression patterns of the other transcripts (*PvDREB2A*, *PvDREB5A*, and *PvDREB6B*) is not appropriate. *PvDREB1F* showed low basal levels of expression in control conditions in all plant organs. The other genes possessed much higher amounts of transcripts in control plants (Supplementary File 14). In addition, the high values of expression of *PvDREB1F* indicate a rapid and greater accumulation of transcripts after stress, which still are lower than those detected for *PvDREB2A*, *PvDREB5A*, and *PvDREB6B*. As a result, *PvDREB1F* remains as the gene with the highest inducibility in this study, but with a lower amount of transcript levels than the other genes.

*PvDREB2A* was predominantly downregulated under the conditions and tissues used in this study. Its expression was induced localized to the stem under dehydration, although some increases in relative amounts of transcripts were observed for cold treatment as well. This is distinct from *GmDREB2A*, from soybean, that had high inducibilities in aerial tissues (including leaves) under stresses caused by high and low temperature, dehydration, and high salinity [85]. In *Arabidopsis*, its inducibility was detected under salinity and drought stress [9]. Overall, in *Arabidopsis*, members of the A-2 subgroup have been mostly characterized by their response to osmotic stresses, especially to dehydration and salinity [3, 5, 6]. Their engineering into other species has increased drought tolerance, even in field conditions [86].

The other genes, *PvDREB5A* and *PvDREB6B*, were stress-inducible as reported in literature for members of subgroups A-5 and A-6 [3]. *PvDREB5A*'s inducibility by all treatments was similar to that of another A-5 member from soybean, *GmDREB2* [20]. Somewhat similar inducibility patterns were detected for *PvDREB6B* in related genes such as *GmDREBb* [19], *AtRAP2.4*, and *AtRAP2.4B* [17].

Gene expression was dependent on the time period of stress as well as the location in plants. In general, fast responses were observed for all genes after stress induction. It is a typical behavior of *DREB* genes, as observed with *AtDREB1A* and *AtDREB1B*, whose transcripts rapidly augmented after only 15 minutes of exposure to low temperature. Some other genes present slower responses such as *AtDREB1C*, with significant accumulation of transcripts only after 2.5 hours under cold treatment [87]. Transcript accumulation has also been shown to vary between roots and leaves, such as *AtDREB1*, more frequent in roots under salinity.

Another critical factor for the analysis of expression of *PvDREB* genes is the developmental stage of plants. Stress treatments were applied at the V_3_ stage, in which plant metabolism is concentrated in plant growth and investments in the leaf area for photosynthesis. It is one of the critical stages, when plants are highly sensitive to abiotic stresses. In comparison, FPKM values retrieved from Phytozome for the four transcripts (*PvDREB1F*, *PvDREB2A*, *PvDREB5A*, and *PvDREB6B*) showed different levels of expression in stem, root, and leaves. Young trifoliolates exhibited low expression in comparison to most of the other tissues analyzed by RNA-Seq.

Furthermore, our results showed some differences in expression values among genotypes under the same stress treatments. It is worthy to note the expression profile of *PvDREB2A* of IAC-Carioca 80SH in relation to the other cultivars under PEG (dehydration) treatment. In leaves, the relative expression of *PvDREB2A* was significantly increased in IAC-Carioca 80SH, while all the other genotypes (BAT 93, Jalo EEP558, BAT 477, and RAB 96) had transcript levels relatively diminished to their control. In roots under PEG, *PvDREB2A* showed inducibility in all genotypes, but the highest relative change was detected in IAC-Carioca 80SH, after the three-hour-stress period. A higher relative expression of *PvDREB2A* was also found in IAC-Carioca 80SH in comparison to BAT 477 in previous work studying the effects of water deprivation [61]. A subtractive library showed differential expression of *DREB2a*, as symbolized then. With qPCR analysis, the expression of the gene was relatively increased in IAC-Carioca 80SH compared to BAT 477 [61]. Such comparison, however, should be made with caution, since the treatments of Recchia et al. [61] were applied in greenhouse conditions and our experiments were conducted in controlled chambers within a few hours. Either way, the study reiterates that different mechanisms of the genotypes might determine gene expression. Further examination is required to elucidate the particularities of each genotype.

Comparisons among genotypes might be useful to encounter genes possibly associated with stress tolerance in common bean. In the same manner, genotypes with contrasting expression profiles might be used for further characterization of the regulation patterns of *DREB* genes in different genetic backgrounds. Based on the number of genotypes in our study, however, we could not draw a correlation profile to determine the direct association between genotype tolerance level to their gene expression. The imminent conclusion from our experiments is that the gene expression was modulated in a temporal-, tissue-, and genotype-dependent configuration. A correlation between *DREB* expression and abiotic stress adaptation requires more experiments. Moreover, such a study would probably consider a wider set of genotypes, preferably from a wild background, in order to establish accurate correlations.

Overall, this study opens the possibility of working with *PvDREB* loci under multiple approaches. We identified several *PvDREB* genes with different structures, coding for proteins with distinctive motifs that can be explored to understand their function. Their annotation suggested all sequences are transcription factors involved in stress responses, but experimental analyses need to be performed for proving their function. With their chromosomal location, molecular marker studies such as with SNP might be able to identify molecular signatures associated with traits of interest in common bean. As *DREB* genes are inherently involved with abiotic stress regulation, further research should bring enormous contributions to improve common bean varieties. Adverse conditions of the diverse environments in which beans are grown might be severely intensified, and all genomic resources available come to help in the design of proper breeding and engineering strategies.

## 5. Conclusions

In this work, we catalogued the *DREB* gene subfamily in common bean. In total, 54 *DREB* genes were defined according to multiple particularities. All genes fitted six main subgroups (A-1 to A-6) according to previous reports for other model species. Four genes were defined, and their expression profiles were addressed under the effect of abiotic stress sources (dehydration, salinity, and low temperature). The major inducibility factors of *PvDREB1F* (dehydration, salinity, and low temperature), *PvDREB2A* (dehydration and cold), *PvDREB5A* (dehydration, salinity, and low temperature), and *PvDREB6B* (dehydration and cold) were determined. However, relative expression levels of each transcript were time-, tissue-, and genotype-modulated. Our categorization along with the isolation and gene expression profile of *PvDREB* genes provides insights for further studies aimed at the improvement of abiotic stress tolerance in common bean.

## Figures and Tables

**Table 1:** Single-nucleotide polymorphisms, polymorphic information content (PIC), and number of haplotypes within the sequences of four *PvDREB* genes, based on 17 common bean genotypes.

Gene	Length of the amplified sequences (bp)	Number of SNP^∗^	Average PIC	Number of haplotypes	Number of nonsynonymous substitutions^∗∗^ caused by SNP and INDEL
*PvDREB1F*	ORF: 882, intron: 288	10^∗∗∗^	0.432	5	-^∗∗∗∗∗^
*PvDREB2A*	ORF: 600, intron: 650	9^∗∗∗^	0.412	6	1
*PvDREB5A*	ORF: 474, 483^∗∗∗∗^	1	0.475	2	0 (SNP) and 3 (INDEL)
*PvDREB6B*	ORF: 957	18	0.408	5	9

^∗^SNPs from G40111 were not included for comparisons. ^∗∗^Number of amino acids changed due point mutations or frame shifts and INDEL. ^∗∗∗^Frame shifts were not accounted. ^∗∗∗∗^The size of the ORF was different between Andean (474 bp) and Mesoamerican (483 bp) due to an INDEL not accounted here. ^∗∗∗∗∗^Not analyzed since no complete sequences were obtained.

## Data Availability

The sequences of the four genes isolated in this work were deposited to GenBank, with the following ID codes: GenBank KX151399, GenBank KX151398, GenBank KX151397, and GenBank KX147642. Their variants in other genotypes can be found in GenBank as well, associated to the same nomenclature of each gene. Other sequence information, pictures of the experiments performed, and general data obtained are presented in the supplementary materials. Several analyses were also based on sequences deposited to the public databases Phytozome (https://phytozome.jgi.doe.gov/pz/portal.html) and TAIR (https://www.arabidopsis.org/). Any further details on the data can be obtained directly with the corresponding author.

## Supplementary Materials

Supplementary 1 Supplementary File S1: BLASTp annotation file of all AP2-containing sequences from common bean. All protein sequences were retrieved from Phytozome.

Supplementary 2 Supplementary File S2: phylogenetic tree.

Supplementary 3 Supplementary File S3: microarray expression profile (upregulated is red and downregulated is green) of DREB genes of Arabidopsis thaliana, under several abiotic treatments, generated by the Genevestigator analysis tool. Codes above heat maps represent the following genes: AT2G40220—gene ABI4, AT5g11590—gene TINY2, AT1G46788—gene RAP2.1, AT2G36900—gene RAP2.10, AT2G40340—gene DREB2C, AT3G11020—gene DREB2B, AT5GO5410—gene DREB2A, AT4G25480—gene DREB1A/CBF3, AT5G51990—gene DREB1D/CBF4, AT4G25490—gene DREB1B/CBF1, AT4G25470—gene DREB1C/CBF2, AT1G22190—gene RAP2.4, and AT1G78080—gene RAP2.4/WIND1.

Supplementary 4 Supplementary File S4: primers designed for PvDREB cloning and RT-qPCR analyses.

Supplementary 5 Supplementary File S5: experiment pictures showing BAT 477 plants submitted to four abiotic stresses in controlled conditions: dehydration (PEG 10%), salinity (NaCl 250 mM), low temperature (4°C), and abscisic acid (ABA 100 *μ*M). The picture shows plants at different time periods of stress induction.

Supplementary 6 Supplementary File S6: protein conserved motifs found within 54 putative DREB proteins from common bean.

Supplementary 7 Supplementary File S7: chromosomal distance (bp) among all the putative DREB genes, indicating possible duplication events. From the strict concept adopted in this study, six potential recent duplication events were described, but other genes are nearby each other, which could be an indication of previous duplication with chromosomal rearrangements and new variation among genes.

Supplementary 8 Supplementary File S8: Blast2Go annotation file.

Supplementary 9 Supplementary File S9: neighbor-joining phylogenetic tree showing the main genes from each of the DREB subgroups (A-1 to A-6). Protein sequences DREB2A and DREB2B are included in the analysis. DREB2A is actually within subgroup A-6, and its name has been replaced by PvDREB6B, which shows the equivalent sequence (although with no full coverage when aligned).

Supplementary 10 Supplementary File S10: relative position of SNP markers of the BARCBean 6k BeadChip (Illumina) from DREB genes in the common bean genome.

Supplementary 11 Supplementary File S11: temporal analysis of the effects of four abiotic stresses on the relative water content (RWC) and enzyme activity (catalase) of BAT 477 leave samples. A—RWC (previous page) and B—catalase activity. Distinct letters over column bars indicate significant differences according to Tukey's test (*P* < 0.05).

Supplementary 12 Supplementary File S12: effects of four stress treatments on the relative water content and the percentage of reduction in water content (A) and catalase activity (B) of five common bean genotypes. Catalase activity is shown on next page. The genotypes differ in their levels of tolerance to abiotic stresses. In general, they have been researched for their drought tolerance levels: RAB96—sensitive, Carioca 80SH (sensitive or moderately sensitive), BAT 477 (tolerant), BAT 93 (we categorized as tolerant), and Jalo EEP558 (we categorized as sensitive). Distinct letters over or below the column bars indicate significant differences according to Tukey's test (*P* < 0.05).

Supplementary 13 Supplementary File S13: complimentary analysis of protein motif predictions with the MEME tool. Sequences include putative DREB proteins from common bean, Arabidopsis thaliana, and Glycine max.

Supplementary 14 Supplementary File S14: (A) amplification of cDNA samples submitted to no treatment with PvDREB genes reveals differences in the initial amounts of transcripts of each gene. (B) A comparison between amplification of PvDREB1F and the other genes under stress treatments with leaf samples from five genotypes under control (C), dehydration (D), salinity (S), freezing (F), and ABA (A).
