# Supplementary material for: DREB Genes from Common Bean (Phaseolus vulgaris L.) Show Broad to Specific Abiotic Stress Responses and Distinct Levels of Nucleotide Diversity
Source: Int J Genomics. 2019 May 2;2019:9520642. doi: 10.1155/2019/9520642 (PMC6525893; doi:10.1155/2019/9520642)
Supplement: Supplementary 2 — Supplementary File S2: phylogenetic tree. [file 9520642.f2.pdf]

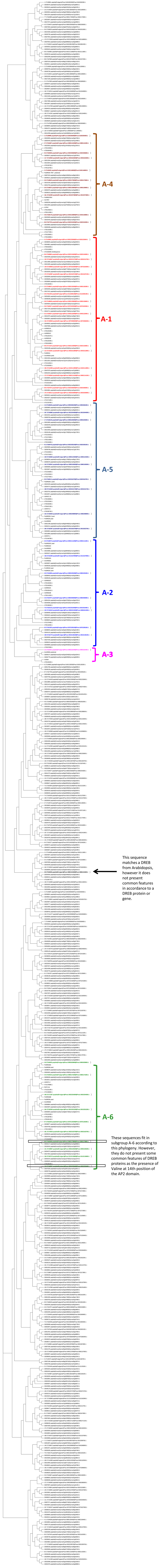
A-4
A-1
A-5
A-2
A-3
This sequence matches a DREB from Arabidopsis, however it does not present common features in accordance to a DREB protein or gene.
A-6
These sequences fit in subgroup A-6 according to this phylogeny. However, they do not present some common features of DREB proteins as the presence of Valine at 14th position of the AP2 domain.
