# Supplementary figures and images for: DREB Genes from Common Bean (Phaseolus vulgaris L.) Show Broad to Specific Abiotic Stress Responses and Distinct Levels of Nucleotide Diversity
Source: Int J Genomics. 2019 May 2;2019:9520642. doi: 10.1155/2019/9520642 (PMC6525893; doi:10.1155/2019/9520642)

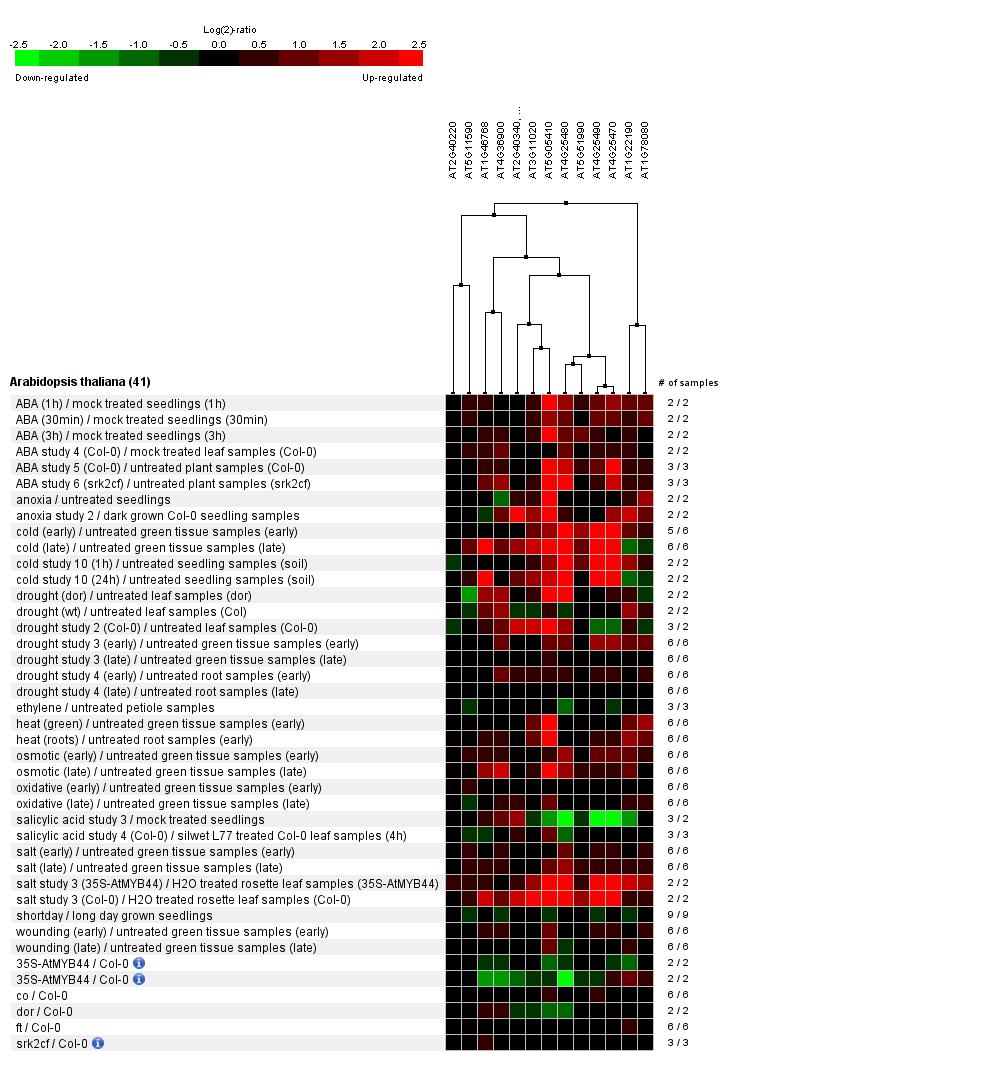

Supplement: Supplementary 3 — Supplementary File S3: microarray expression profile (upregulated is red and downregulated is green) of DREB genes of Arabidopsis thaliana, under several abiotic treatments, generated by the Genevestigator analysis tool. Codes above heat maps represent the following genes: AT2G40220—gene ABI4, AT5g11590—gene TINY2, AT1G46788—gene RAP2.1, AT2G36900—gene RAP2.10, AT2G40340—gene DREB2C, AT3G11020—gene DREB2B, AT5GO5410—gene DREB2A, AT4G25480—gene DREB1A/CBF3, AT5G51990—gene DREB1D/CBF4, AT4G25490—gene DREB1B/CBF1, AT4G25470—gene DREB1C/CBF2, AT1G22190—gene RAP2.4, and AT1G78080—gene RAP2.4/WIND1. [file 9520642.f3.jpg]

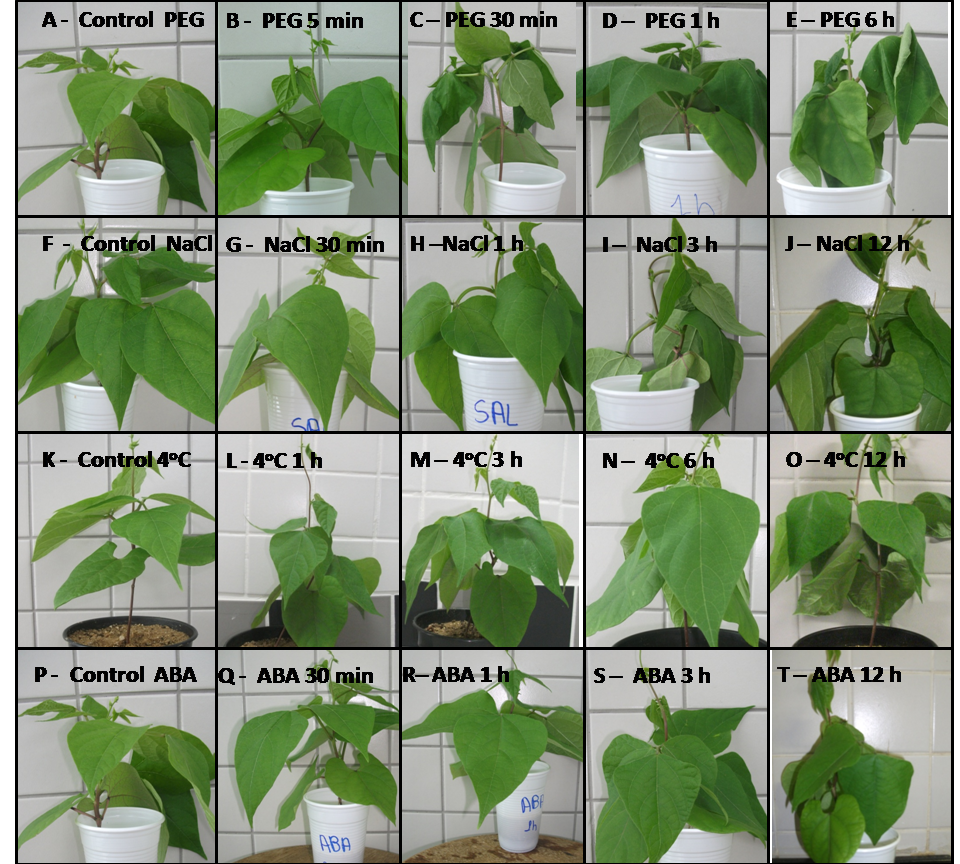

Supplement: Supplementary 5 — Supplementary File S5: experiment pictures showing BAT 477 plants submitted to four abiotic stresses in controlled conditions: dehydration (PEG 10%), salinity (NaCl 250 mM), low temperature (4°C), and abscisic acid (ABA 100 μM). The picture shows plants at different time periods of stress induction. [file 9520642.f5.tif]

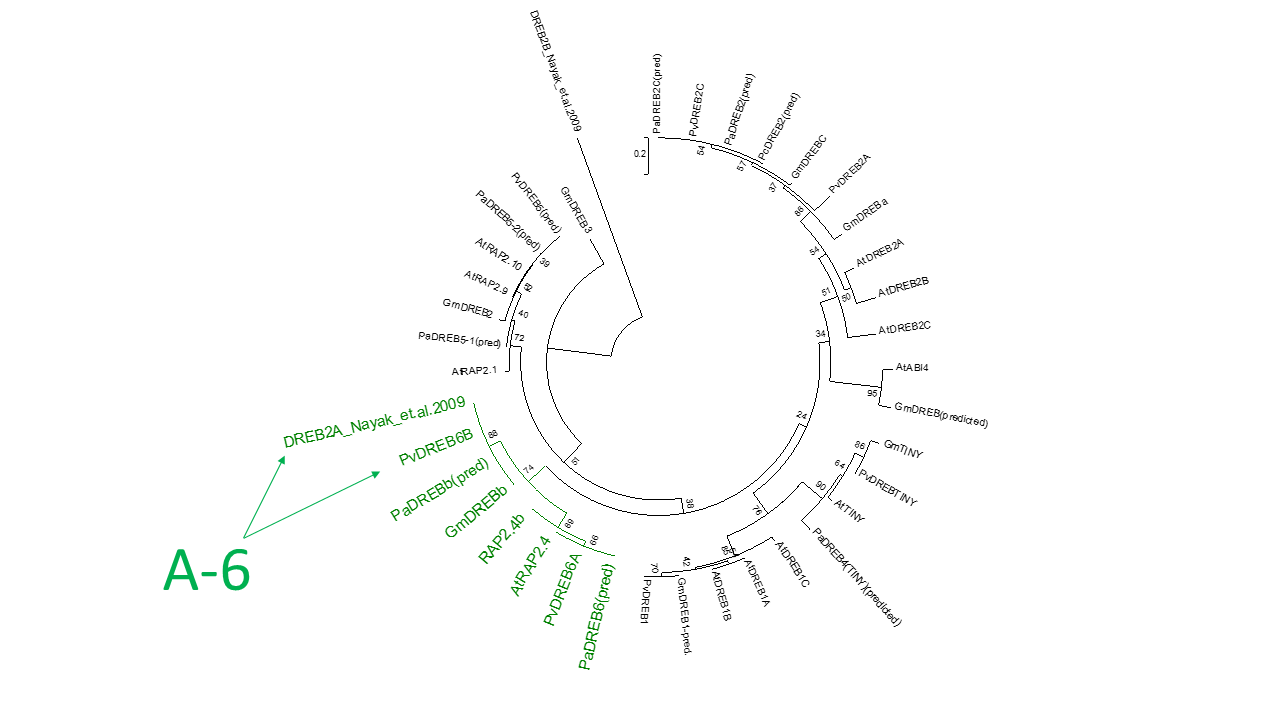

Supplement: Supplementary 9 — Supplementary File S9: neighbor-joining phylogenetic tree showing the main genes from each of the DREB subgroups (A-1 to A-6). Protein sequences DREB2A and DREB2B are included in the analysis. DREB2A is actually within subgroup A-6, and its name has been replaced by PvDREB6B, which shows the equivalent sequence (although with no full coverage when aligned). [file 9520642.f9.tif]

A

## RWC

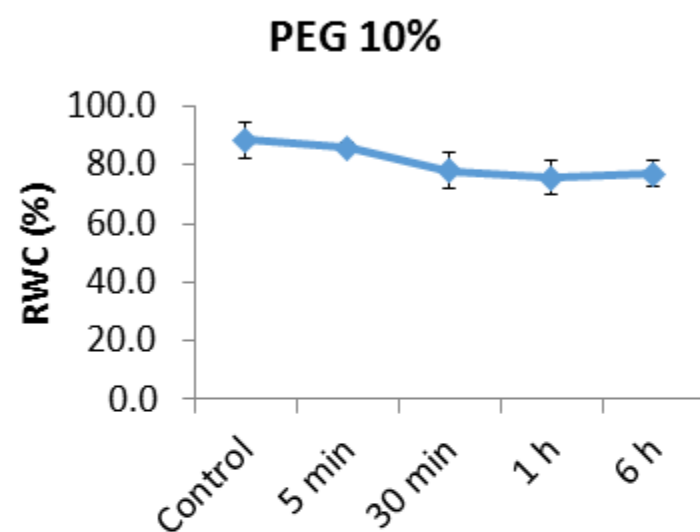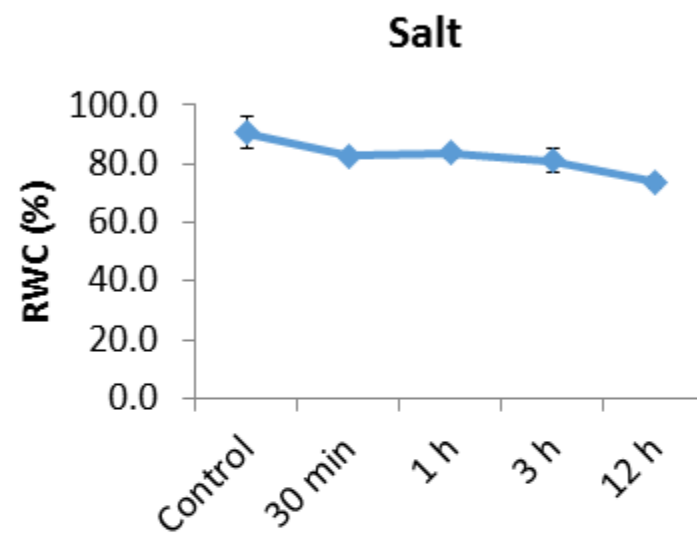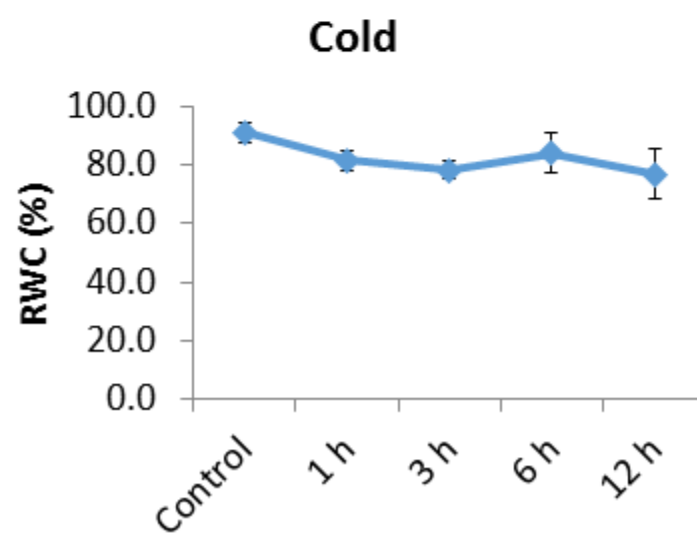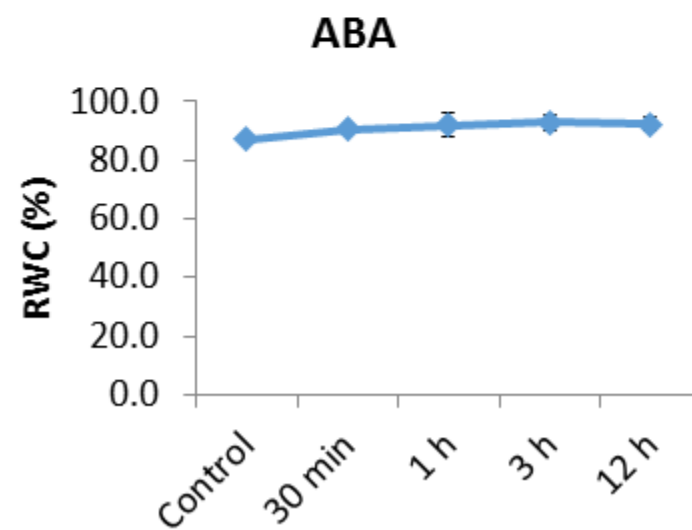

B

## CATALASE ACTIVITY

PEG 10%

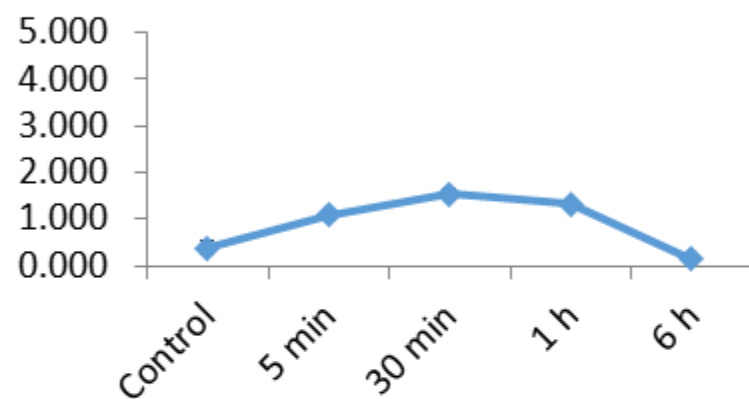

Salt

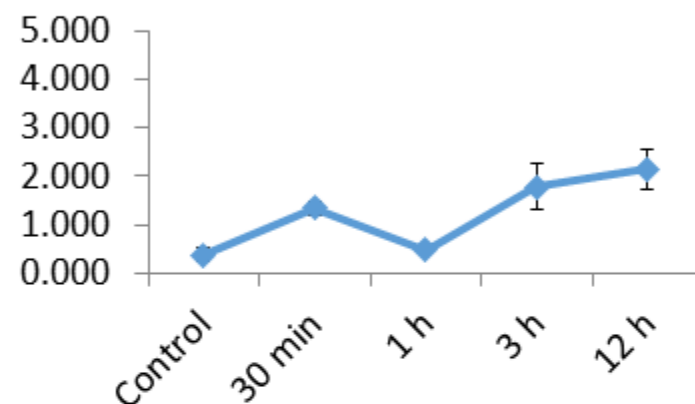

Cold

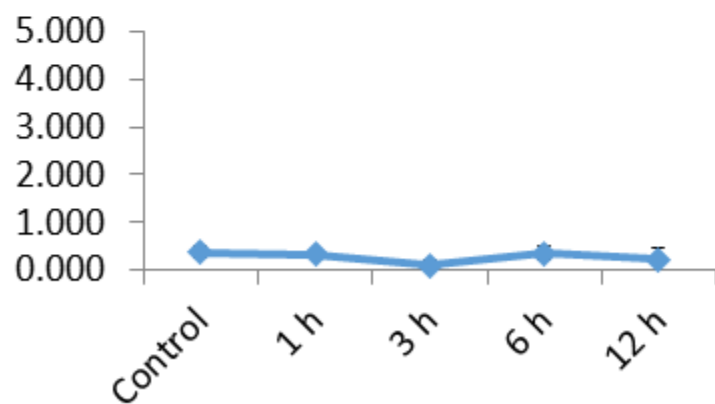

ABA

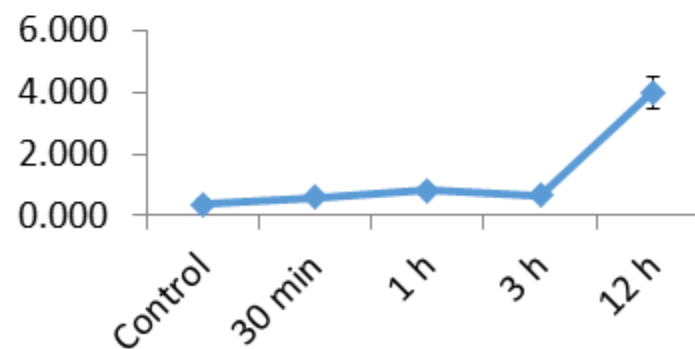

Supplement: Supplementary 11 — Supplementary File S11: temporal analysis of the effects of four abiotic stresses on the relative water content (RWC) and enzyme activity (catalase) of BAT 477 leave samples. A—RWC (previous page) and B—catalase activity. Distinct letters over column bars indicate significant differences according to Tukey's test (P < 0.05). [file 9520642.f11.pdf]

A

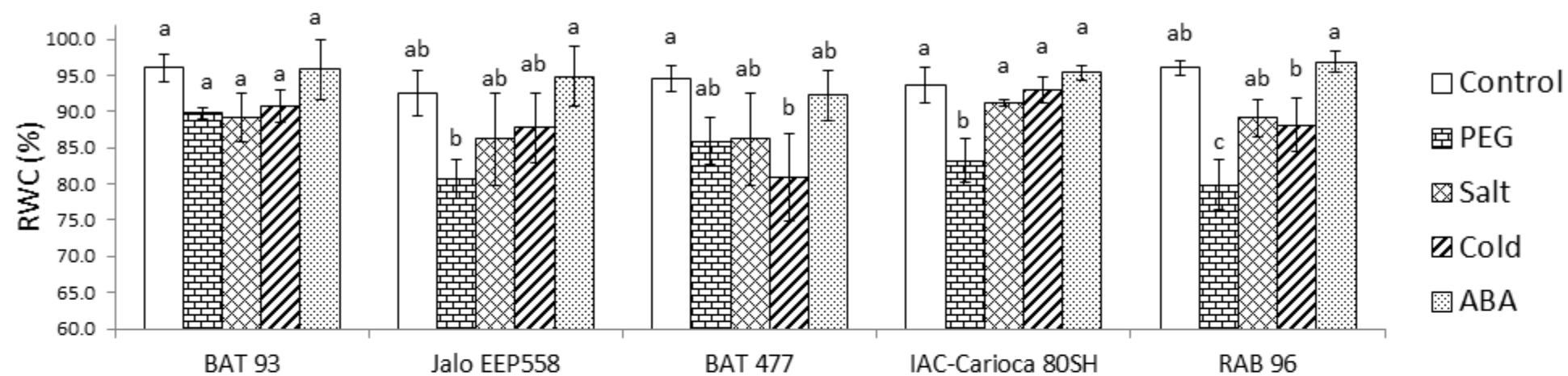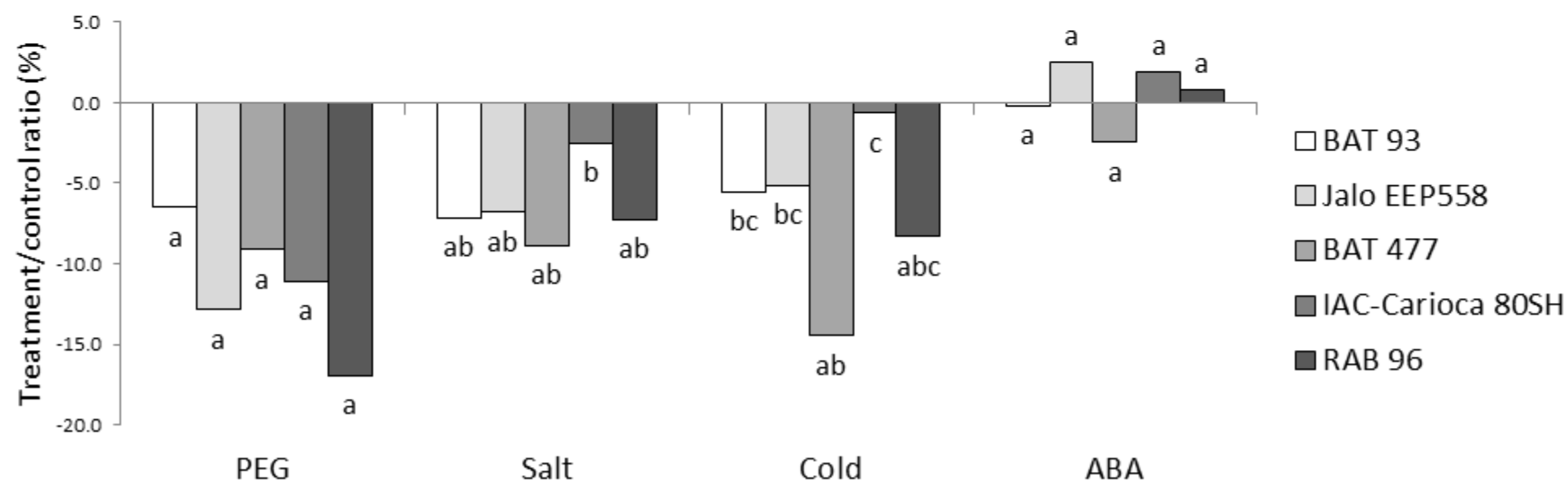

B

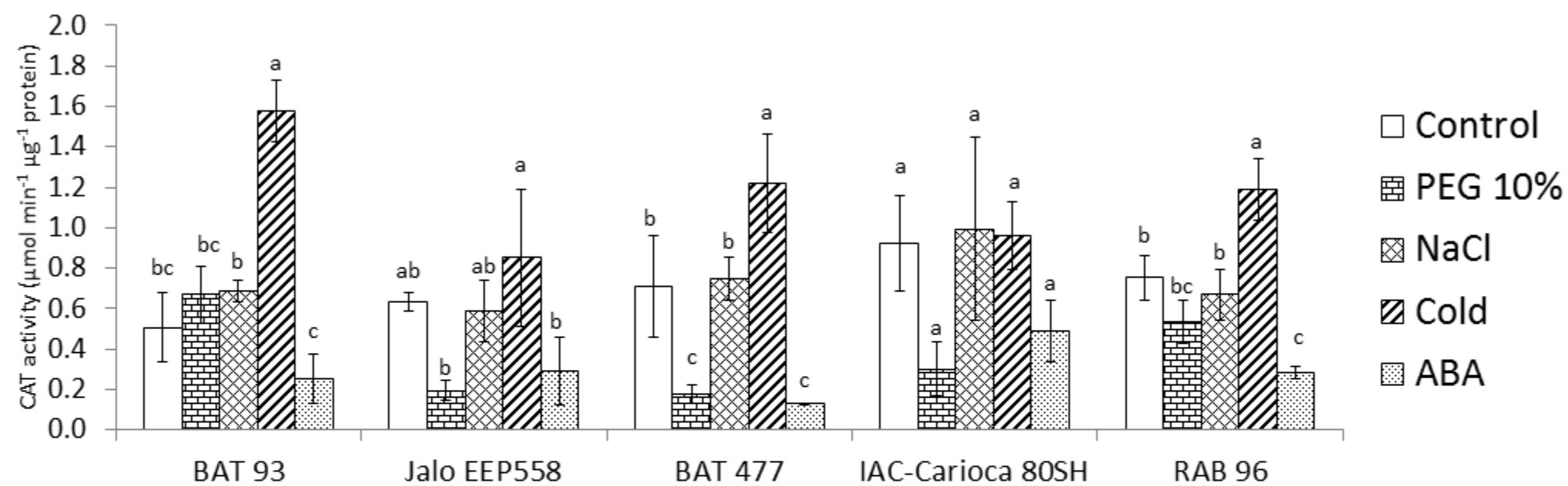

Supplement: Supplementary 12 — Supplementary File S12: effects of four stress treatments on the relative water content and the percentage of reduction in water content (A) and catalase activity (B) of five common bean genotypes. Catalase activity is shown on next page. The genotypes differ in their levels of tolerance to abiotic stresses. In general, they have been researched for their drought tolerance levels: RAB96—sensitive, Carioca 80SH (sensitive or moderately sensitive), BAT 477 (tolerant), BAT 93 (we categorized as tolerant), and Jalo EEP558 (we categorized as sensitive). Distinct letters over or below the column bars indicate significant differences according to Tukey's test (P < 0.05). [file 9520642.f12.pdf]

A-1

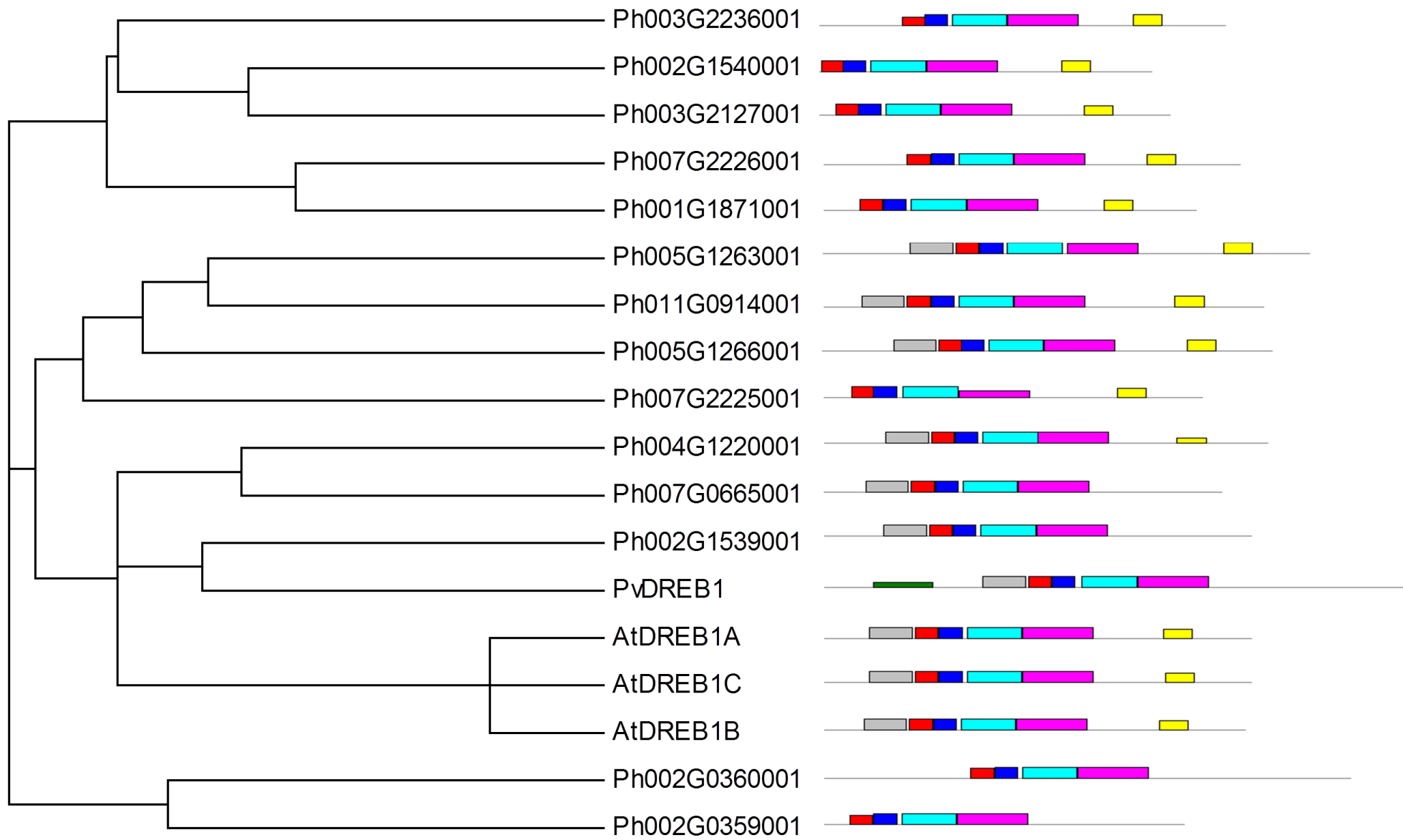

0.30 0.25 0.20 0.15 0.10 0.05 0.00

A-2

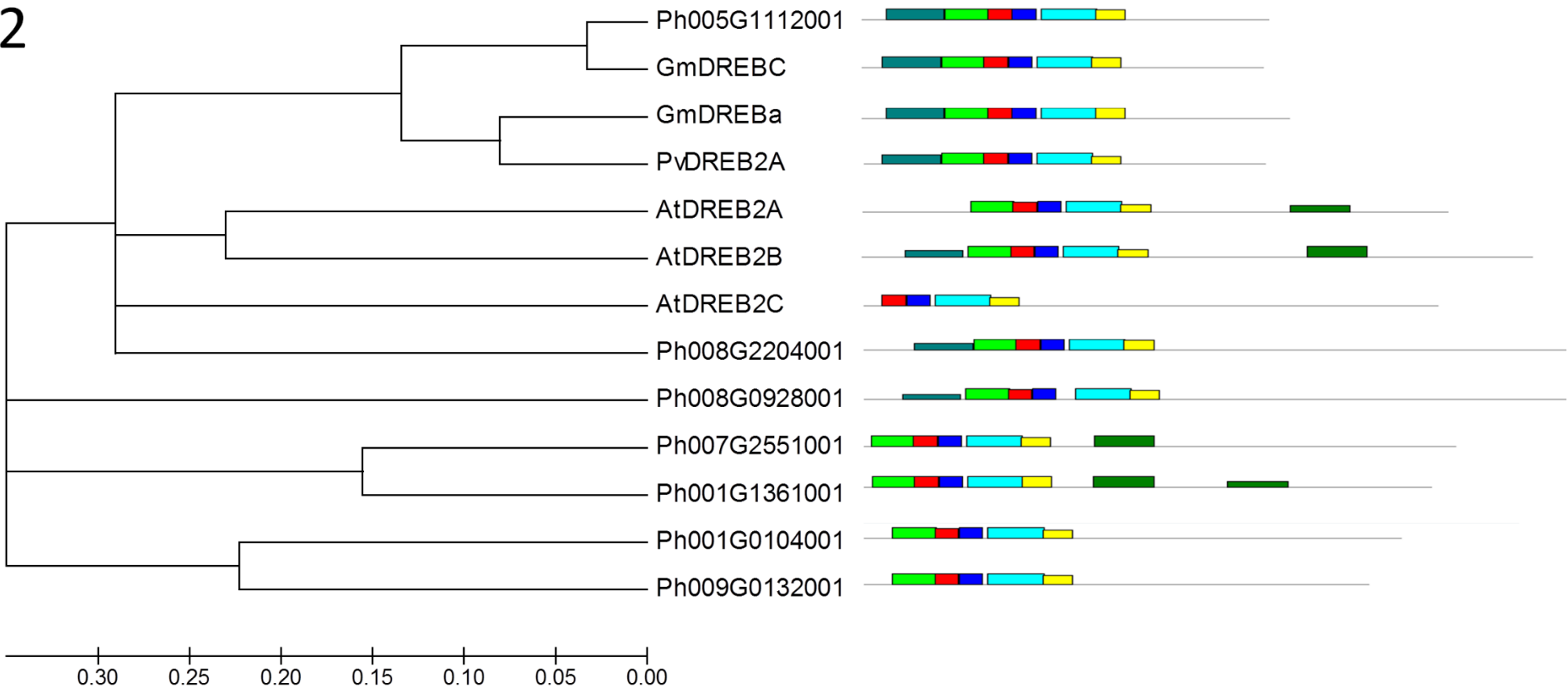

A-3

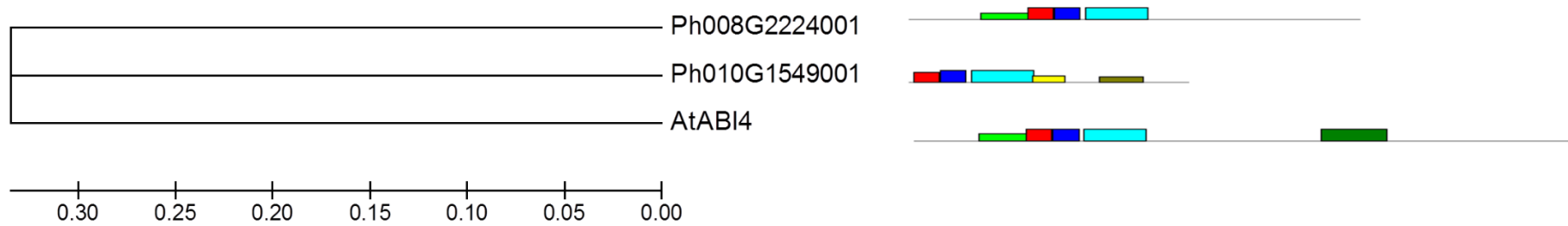

A-4

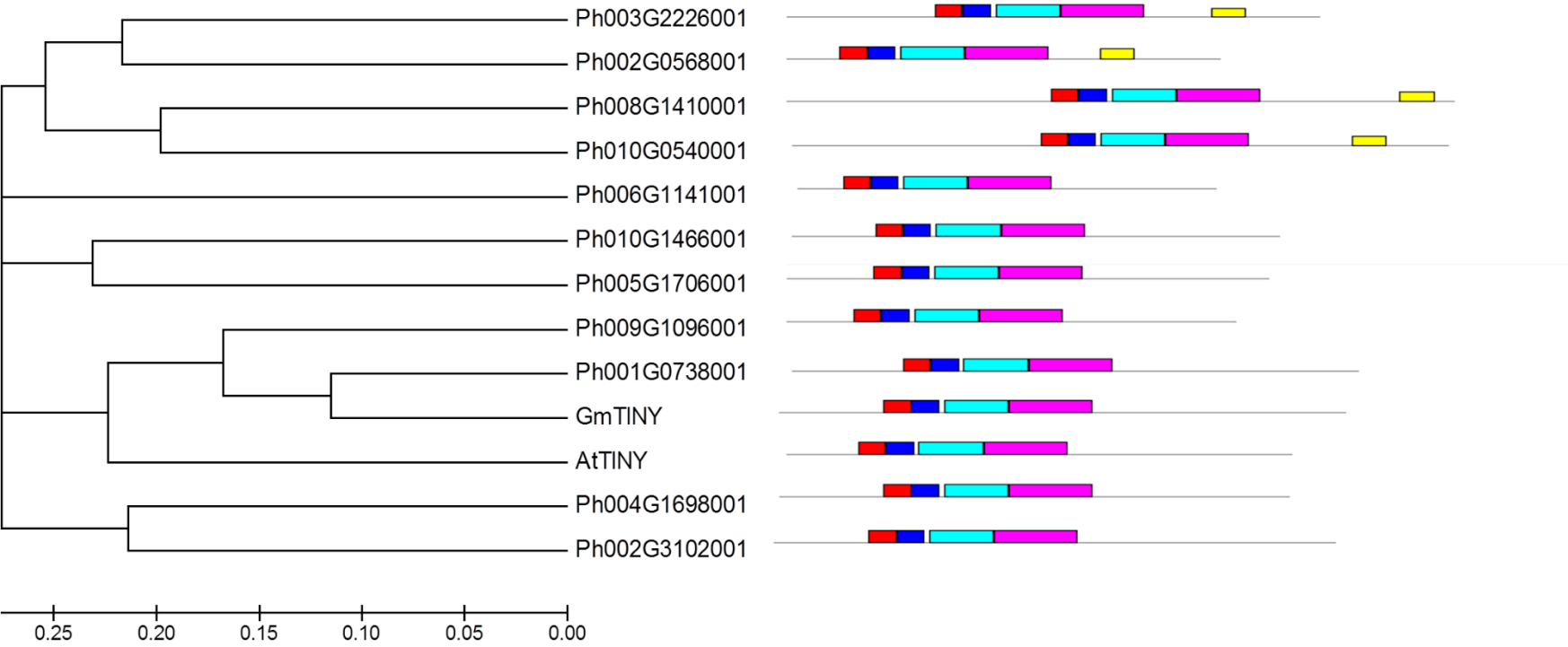

A-5

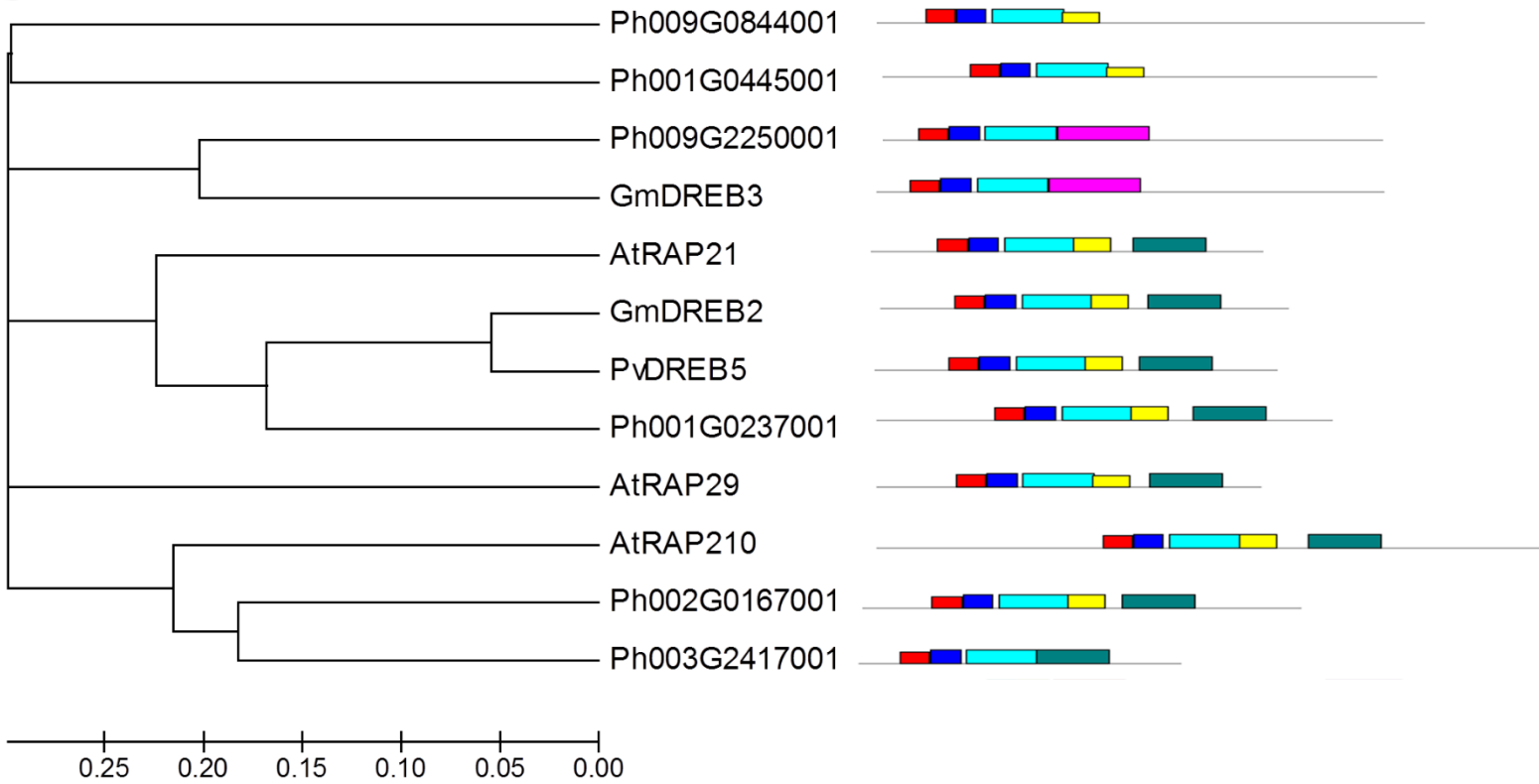

A-6

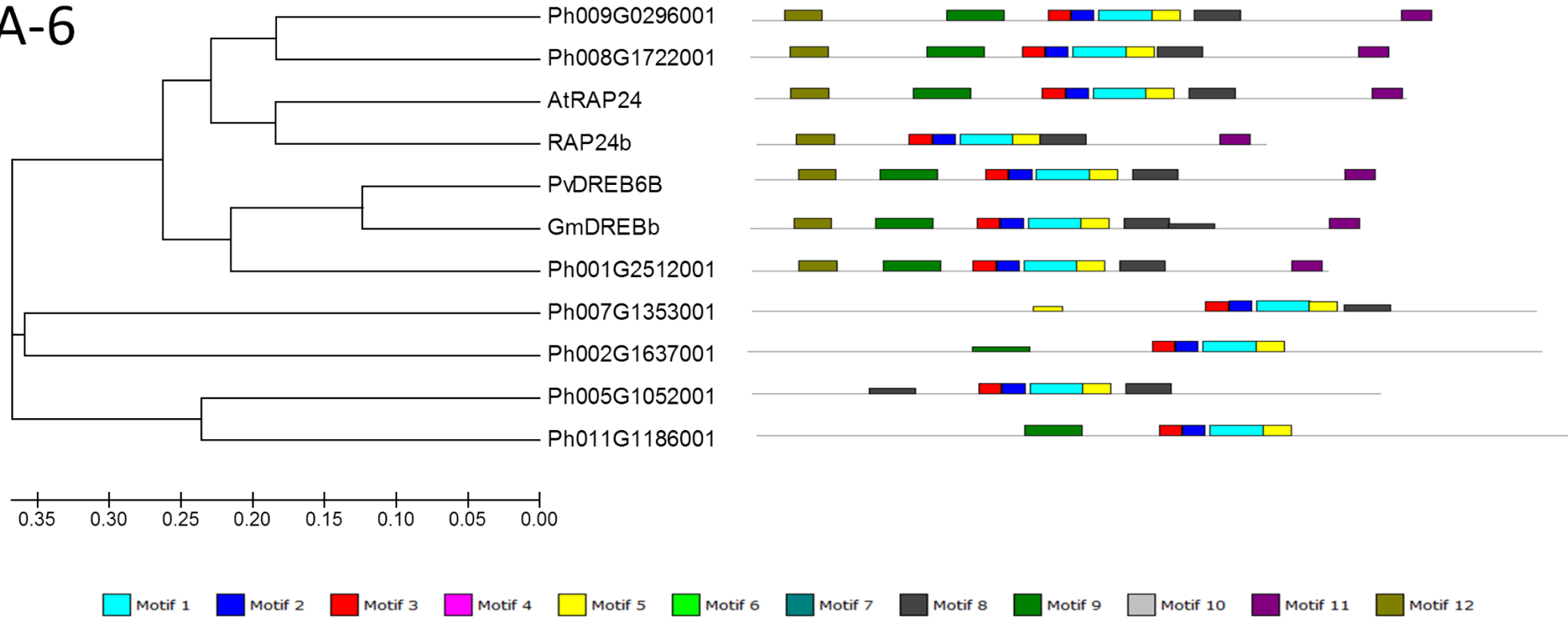

Supplement: Supplementary 13 — Supplementary File S13: complimentary analysis of protein motif predictions with the MEME tool. Sequences include putative DREB proteins from common bean, Arabidopsis thaliana, and Glycine max. [file 9520642.f13.pdf]

A

cDNA of leaf control sample (no stress)

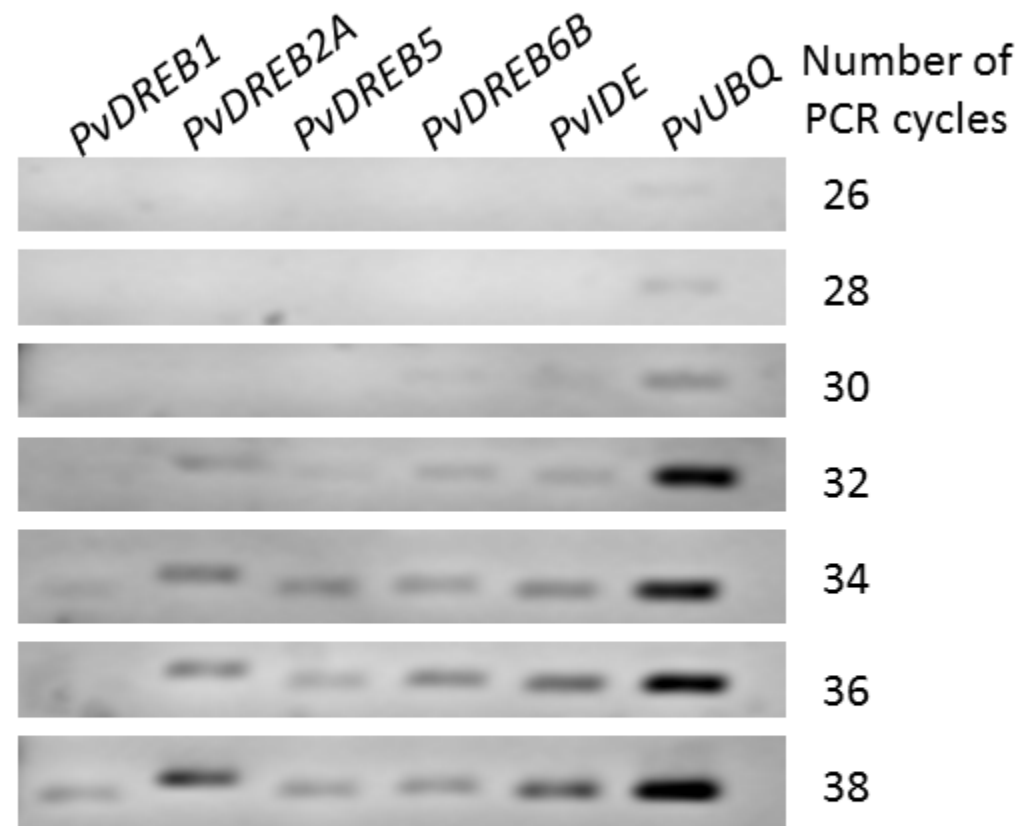

**B**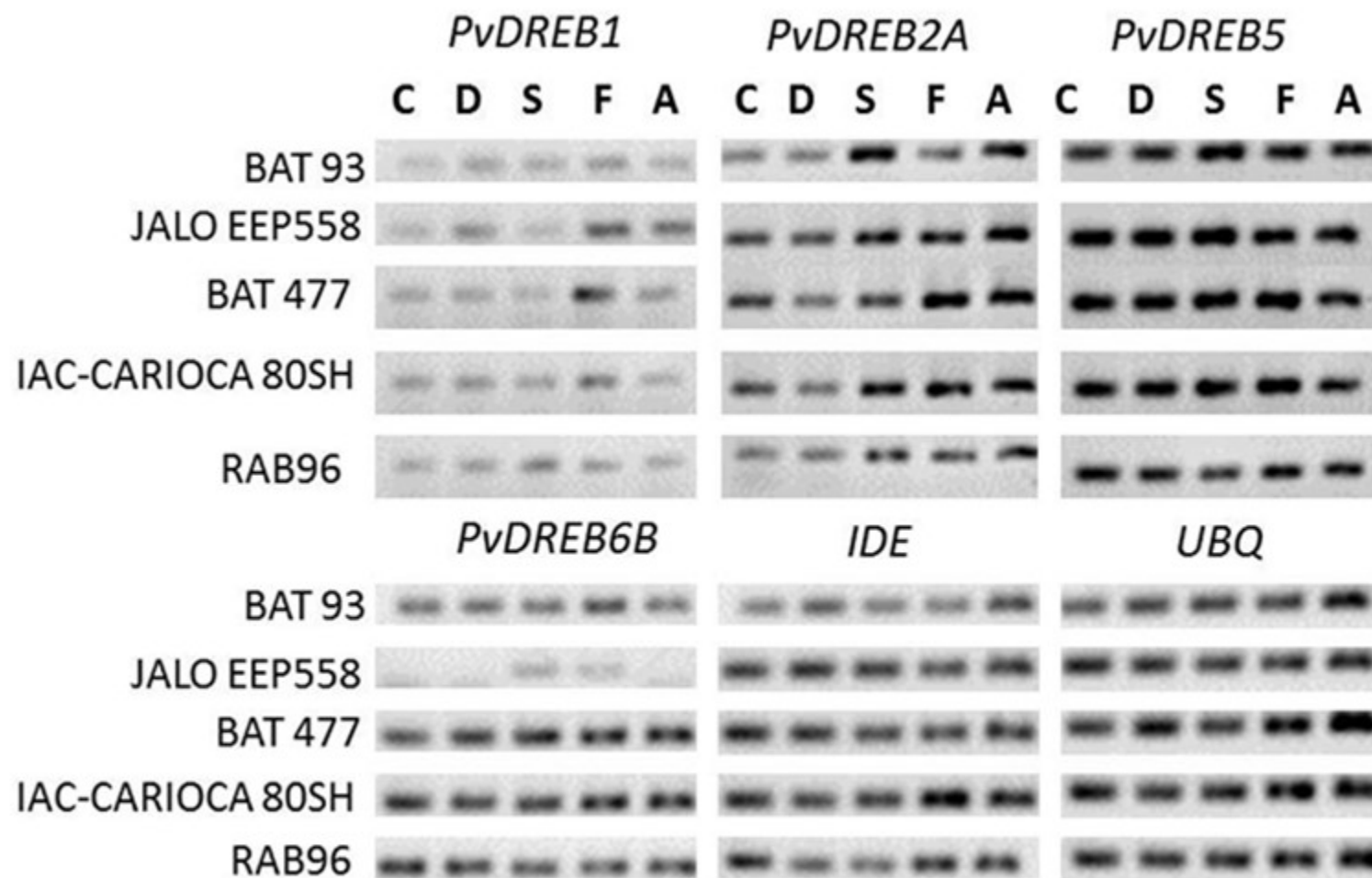

Supplement: Supplementary 14 — Supplementary File S14: (A) amplification of cDNA samples submitted to no treatment with PvDREB genes reveals differences in the initial amounts of transcripts of each gene. (B) A comparison between amplification of PvDREB1F and the other genes under stress treatments with leaf samples from five genotypes under control (C), dehydration (D), salinity (S), freezing (F), and ABA (A). [file 9520642.f14.pdf]
