# Supplementary material for: DREB Genes from Common Bean (Phaseolus vulgaris L.) Show Broad to Specific Abiotic Stress Responses and Distinct Levels of Nucleotide Diversity
Source: Int J Genomics. 2019 May 2;2019:9520642. doi: 10.1155/2019/9520642 (PMC6525893; doi:10.1155/2019/9520642)
Supplement: Supplementary 10 — Supplementary File S10: relative position of SNP markers of the BARCBean 6k BeadChip (Illumina) from DREB genes in the common bean genome. [file 9520642.f10.docx]

**Supplementary File 10** Relative position of SNP markers of the BarcBean 6k BeadChip (Illumina) from *DREB* genes in common bean genome.

| ***DREB* genes location** | | | | ***SNP location*** | | **Distance from SNP to the gene** | |
| --- | --- | --- | --- | --- | --- | --- | --- |
| **Chromosome** | **Initial position (bp)** | **Final position (bp)** | **Phytozome ID** | ***Chromosome*** | ***Specific position*** | **Distance from initiation site (kb)** | **Distance from end (kb)** |
| Chr01 | 931454 | 932855 | Phvul.001G010400 | Chr01 | 927076 | -4.378 | -5.779 |
| Chr01 | 2101326 | 2102436 | Phvul.001G023700 | Chr01 | 2153411 | 52.085 | 50.975 |
| Chr01 | 4680371 | 4681060 | Phvul.001G044500 | Chr01 | 4623673 | -56.698 | -57.387 |
| Chr01 | 10050549 | 10051844 | Phvul.001G073800 | Chr01 | 10059902 | 9.353 | 8.058 |
| Chr01 | 38195399 | 38196241 | Phvul.001G136100 | Chr01 | 38347891 | 152.492 | 151.650 |
| Chr01 | 45289609 | 45290175 | Phvul.001G187100 | Chr01 | 45286535 | -3.074 | -3.640 |
| Chr01 | 50886895 | 50888163 | Phvul.001G251200 | Chr01 | 50546984 | -339.911 | -341.179 |
| Chr02 | 1837177 | 1838211 | Phvul.002G016700 | Chr02 | 1837703 | 0.526 | -0.508 |
| Chr02 | 3499002 | 3499535 | Phvul.002G035100 | Chr02 | 3448505 | -50.497 | -51.030 |
| Chr02^1^ | 3545434 | 3545982 | Phvul.002G035900 | Chr02 | 3570055 | 24.621 | 24.073 |
| Chr02^1^ | 3561530 | 3562521 | Phvul.002G036000 | Chr02 | 3570055 | 8.525 | 7.534 |
| Chr02 | 5810982 | 5811876 | Phvul.002G056800 | Chr02 | 5808344 | -2.638 | -3.532 |
| Chr02^2^ | 29528341 | 29528991 | Phvul.002G153900 | Chr02 | 29516525 | -11.816 | -12.466 |
| Chr02^2^ | 29572213 | 29572719 | Phvul.002G154000 | Chr02 | 29516525 | -55.688 | -56.194 |
| Chr02 | 30545248 | 30546790 | Phvul.002G163700 | Chr02 | 30457680 | -87.568 | -89.110 |
| Chr02 | 42080727 | 42082576 | Phvul.002G254500 (*PvDREB6B*) | Chr02 | 42070533 | -10.194 | -12.043 |
| Chr02 | 47177131 | 47178333 | Phvul.002G310200 | Chr02 | 46918900 | -258.231 | -259.433 |
| Chr03 | 42784293 | 42784829 | Phvul.003G212700 | Chr03 | 42790079 | 5.786 | 5.250 |
| Chr03 | 42804542 | 42805711 | Phvul.003G212800 (*PvDREB1*) | Chr03 | 42803103 | -1.439 | -2.608 |
| Chr03 | 44044998 | 44045690 | Phvul.003G222600 | Chr03 | 43979309 | -65.689 | -66.381 |
| Chr03 | 44193652 | 44195231 | Phvul.003G223600 | Chr03 | 44265574 | 71.922 | 70.343 |
| Chr03 | 46504117 | 46504556 | Phvul.003G241700 | Chr03 | 46585905 | 81.788 | 81.349 |
| Chr04 | 39326716 | 39327951 | Phvul.004G122000 | Chr04 | 39341575 | 14.859 | 13.624 |
| Chr04 | 45126736 | 45127899 | Phvul.004G169800 | Chr04 | 45170785 | 44.049 | 42.886 |
| Chr05 | 31203406 | 31204629 | Phvul.005G105200 | Chr05 | 31211857 | 8.451 | 7.228 |
| Chr05 | 32566424 | 32568115 | Phvul.005G111200 | Chr05 | 32370244 | -196.180 | -197.871 |
| Chr05 | 35040940 | 35041680 | Phvul.005G126300 | Chr05 | 35048031 | 7.091 | 6.351 |
| Chr05 | 35099239 | 35100039 | Phvul.005G126600 | Chr05 | 35065924 | -33.315 | -34.115 |
| Chr05 | 39420680 | 39421777 | Phvul.005G170600 | Chr05 | 39426213 | 5.533 | 4.436 |
| Chr06 | 22942518 | 22943272 | Phvul.006G114100 | Chr06 | 22953680 | 11.162 | 10.408 |
| Chr07 | 5928454 | 5929059 | Phvul.007G066500 | Chr07 | 5986656 | 58.202 | 57.597 |
| Chr07 | 33385083 | 33386444 | Phvul.007G135300 | Chr07 | 33621462 | 236.379 | 235.018 |
| Chr07^3^ | 46214380 | 46215168 | Phvul.007G222500 | Chr07 | 46187463 | -26.917 | -27.705 |
| Chr07^3^ | 46221519 | 46222906 | Phvul.007G222600 | Chr07 | 46187463 | -34.056 | -35.443 |
| Chr07 | 49321858 | 49323149 | Phvul.007G255100 | Chr07 | 49327677 | 5.819 | 4.528 |
| Chr08 | 9500856 | 9502893 | Phvul.008G092800 | Chr08 | 9519416 | 18.560 | 16.523 |
| Chr08 | 10466381 | 10466854 | Phvul.008G098900 (*PvDREB5A*) | Chr08 | 10458119 | -8.262 | -8.735 |
| Chr08 | 23916631 | 23917738 | Phvul.008G141000 | Chr08 | 24044227 | 127.596 | 126.489 |
| Chr08 | 42621369 | 42622218 | Phvul.008G165000 | Chr08 | 42612989 | -8.380 | -9.229 |
| Chr08 | 45116054 | 45117855 | Phvul.008G172200 | Chr08 | 45096307 | -19.747 | -21.548 |
| Chr08 | 53286141 | 53288298 | Phvul.008G220400 | Chr08 | 53313550 | 27.409 | 25.252 |
| Chr08 | 53485082 | 53486071 | Phvul.008G222400 | Chr08 | 53651728 | 166.646 | 165.657 |
| Chr09 | 2059353 | 2060102 | Phvul.009G013200 | Chr09 | 1896451 | -162.902 | -163.651 |
| Chr09 | 6536069 | 6538397 | Phvul.009G029600 | Chr09 | 6610194 | 74.125 | 71.797 |
| Chr09 | 13327339 | 13328429 | Phvul.009G084400 | Chr09 | 13305494 | -21.845 | -22.935 |
| Chr09 | 16431520 | 16432907 | Phvul.009G109600 | Chr09 | 16444498 | 12.978 | 11.591 |
| Chr09 | 18302891 | 18303953 | Phvul.009G123300 | Chr09 | 18434928 | 132.037 | 130.975 |
| Chr09 | 33327983 | 33329052 | Phvul.009G225000 | Chr09 | 33353768 | 25.785 | 24.716 |
| Chr10 | 8503366 | 8504597 | Phvul.010G054000 | Chr10 | 8541459 | 38.093 | 36.862 |
| Chr10 | 38090570 | 38091511 | Phvul.010G114900 | Chr10 | 38093855 | 3.285 | 2.344 |
| Chr10 | 41789538 | 41790170 | Phvul.010G146600 | Chr10 | 42153024 | 363.486 | 362.854 |
| Chr11 | 9148323 | 9148991 | Phvul.011G091400 | Chr11 | 9133461 | -14.862 | -15.530 |
| Chr11 | 12892620 | 12894683 | Phvul.011G107800 (*PvDREB2A*) | Chr11 | 12913506 | 20.886 | 18.823 |
| Chr11 | 19321983 | 19324035 | Phvul.011G118600 | Chr11 | 19373588 | 51.605 | 49.553 |

^1, 2, 3^ Pairs of genes represented by the same SNP.
